# Free SepF interferes with recruitment of late cell division proteins

**DOI:** 10.1038/s41598-017-17155-x

**Published:** 2017-12-05

**Authors:** Yongqiang Gao, Michaela Wenzel, Martijs J. Jonker, Leendert W. Hamoen

**Affiliations:** 1 Swammerdam Institute for Life Sciences, University of Amsterdam, O|2 Building, De Boelelaan 1108, 1081 HZ Amsterdam, The Netherlands; 2 MicroArray Department and Integrative Bioinformatics Unit, Swammerdam Institute for Life Sciences, University of Amsterdam, Sciencepark 904, 1098 XH Amsterdam, The Netherlands

## Abstract

The conserved cell division protein SepF aligns polymers of FtsZ, the key cell division protein in bacteria, during synthesis of the (Fts)Z-ring at midcell, the first stage in cytokinesis. In addition, SepF acts as a membrane anchor for the Z-ring. Recently, it was shown that SepF overexpression in *Mycobacterium smegmatis* blocks cell division. Why this is the case is not known. Surprisingly, we found in *Bacillus subtilis* that SepF overproduction does not interfere with Z-ring assembly, but instead blocks assembly of late division proteins responsible for septum synthesis. Transposon mutagenesis suggested that SepF overproduction suppresses the essential WalRK two-component system, which stimulates expression of *ftsZ*. Indeed, it emerged that SepF overproduction impairs normal WalK localization. However, transcriptome analysis showed that the WalRK activity was in fact not reduced in SepF overexpressing cells. Further experiments indicated that SepF competes with EzrA and FtsA for binding to FtsZ, and that binding of extra SepF by FtsZ alleviates the cell division defect. This may explain why activation of WalRK in the transposon mutant, which increases *ftsZ* expression, counteracts the division defect. In conclusion, our data shows that an imbalance in early cell division proteins can interfere with recruitment of late cell division proteins.

## Introduction

Bacterial cell division is initiated by polymerization of the tubulin homologue FtsZ into a ring-like structure at midcell. This so-called Z-ring or protoring provides the scaffold for the late cell division proteins that synthesize the division septum. In many bacteria FtsZ polymers are linked to each other by the protein ZapA^1,2^, and attached to the cell membrane by the peripheral membrane protein FtsA^3,4^. In Gram-positive and cyanobacteria this function is supported by the peripheral membrane protein SepF^5^. Most bacteria contain a transmembrane protein that interacts with FtsZ, e.g. ZipA in many proteobacteria, and EzrA in most firmicutes. However, these proteins do not seem to function as membrane anchors, but fulfill a regulatory role in the assembly of the Z-ring^5–7^. Once the Z-ring is assembled, the so called late cell division proteins are recruited responsible for synthesize of the division septum, such as the peptidoglycan glycosyltransferase FtsW, and the peptidoglycan transpeptidases Pbp2B in *Bacillus subtilis* and FtsI in *Escherichia coli* ^8–12^. Assembly of the late proteins requires the presence of the conserved transmembrane proteins FtsL, DivIC and DivIB in *B. subtilis* and the homologous proteins FtsL, FtsB and FtsQ in *E. coli* ^13–15^. These proteins do not have clear catalytic domains and it is assumed that they play a structural role and somehow regulate the recruitment of late cell division proteins to the Z-ring. In *B. subtilis*, this recruitment is a strongly cooperative process and the absence of one of the late proteins (except for the non-essential protein DivIB) inhibits assembly of all late cell division proteins^16,17^. For a recent review on bacterial cell division see e.g.^18^.

Recently, it was shown that overproduction of SepF in *Mycobacterium smegmatis* interferes with cell division, resulting in filamentous cells^19^. Actinomycetes, including the mycobacteria, lack FtsA, and SepF is presumably the only membrane anchor for the Z-ring in these bacteria^20^. It is therefore probable that a too high concentration of SepF interferes with the formation of Z-rings. We set out to investigate this using the genetically more tractable bacterium *Bacillus subtilis*.

SepF is highly conserved in Gram-positive bacteria and cyanobacteria, and was first discovered to play a role in cell division in *Streptococcus pneumoniae* and *Synechococcus elongates* ^21,22^. Inactivation of *sepF* in these organisms leads to severe cell division defects. Deletion of *sepF* in *B. subtilis* has a more subtle effect, and only with transmission electron microscopy it becomes apparent that the division septum is strongly deformed when SepF is absent^23^. Purified SepF assembles into large and regular protein rings with an inner diameter of about 40 nm, which is close to the average thickness of septa. Electron microscopic analyses suggested that the membrane binding domain, an N-terminally located amphipathic helix, is located inside the SepF ring. When SepF rings are mixed with polymerizing FtsZ, FtsZ polymers become attached to the rings and long tubular structures are formed^24^. Based on these observations, it was postulated that SepF forms arcs on top of the leading edge of nascent septa, thereby attaching FtsZ polymers to the cell membrane and helping them to align parallel to the plane of division^5^.

Because of these characteristics, it is not surprising that overproduction of SepF in *M. smegmatis* causes a cell division defect. We found that SepF overproduction has the same effect in *B. subtilis*. Surprisingly, however, it appeared that the formation of Z-rings was not disturbed when SepF was overproduced. Extensive genetic analyses, including transcriptomics and transposon mutagenesis, show that SepF overproduction blocks the assembly of late cell division proteins and occurs when excess SepF is not interacting with FtsZ. These data indicate that an imbalance in early cell division proteins can interfere with the assembly of late cell division proteins, and may suggest that SepF also plays a role in the recruitment of these proteins.

## Results

### SepF overexpression is lethal in *B. subtilis*

**Figure 1:**
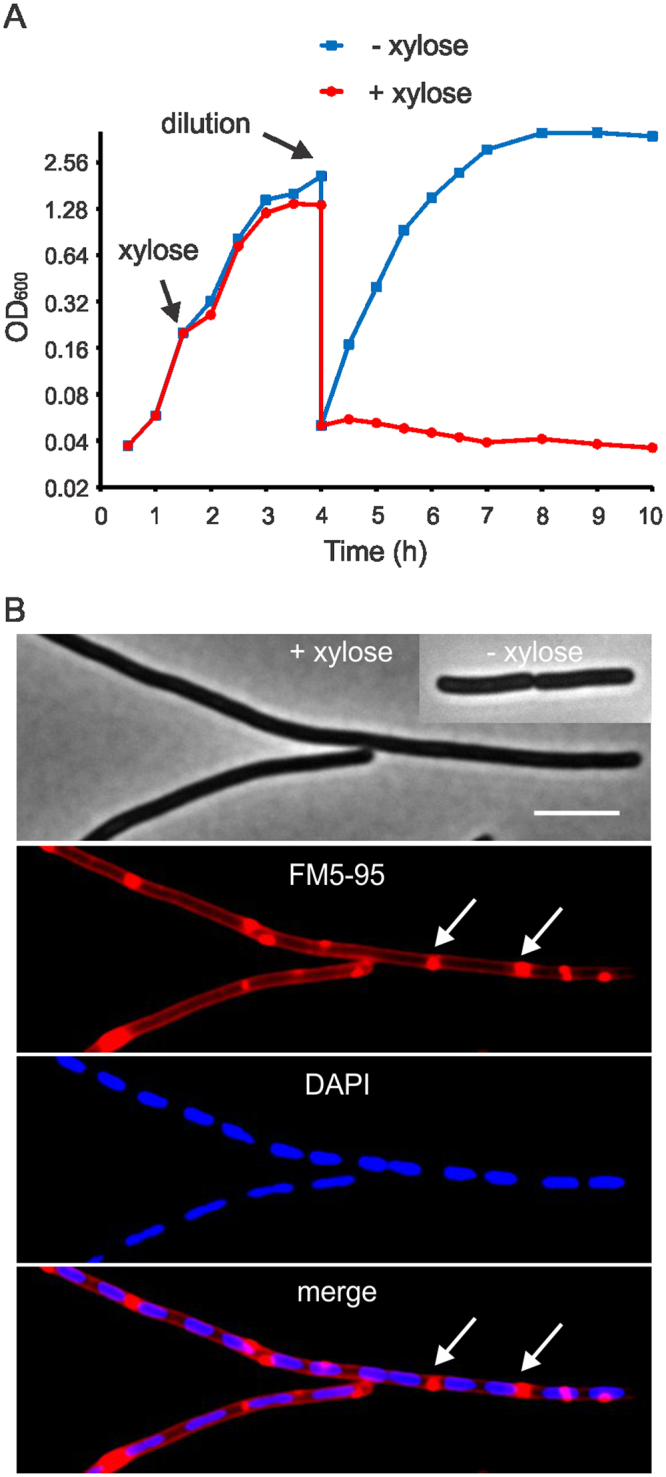
SepF overexpression in *B. subtilis*. (**A**) Growth curve of *B. subtilis* strain GYQ215 containing an extra xylose-inducible *sepF* gene (*amyE*::P*xyl-sepF*) grown with or without 1% xylose. After 3 h induction, cultures were diluted into fresh medium. (**B**) Fluorescence light microscopy images of cells after 3 h induction. Membrane and DNA were stained with FM5-95 and DAPI, respectively. Membrane invaginations are indicated by arrows. Scale bar is 5 µm. More images of membrane stained SepF-overexpressing cells can be found in Fig. S2.

Overexpression of SepF in *M. smegmatis* is lethal and blocks cell division^19^. Why this is the case is not clear. To investigate this in more detail, we tested whether the same phenotype occurred in the more tractable model organism *B. subtilis*. A *B. subtilis* strain was constructed harboring an extra copy of *sepF* under control of the strong xylose-inducible promoter *Pxyl* (strain GYQ215). The presence of 1% xylose resulted in a strong overproduction of SepF (Fig. S1). As shown in Fig. 1, overexpression of SepF in *B. subtilis* resulted in filamentous cells. After approximately 3 h induction, these filamentous cells were unable to grow any further (Fig. 1). This phenotype is comparable to what has been found for *M. smegmatis* ^19^.

### Membrane invaginations

**Figure 2:**
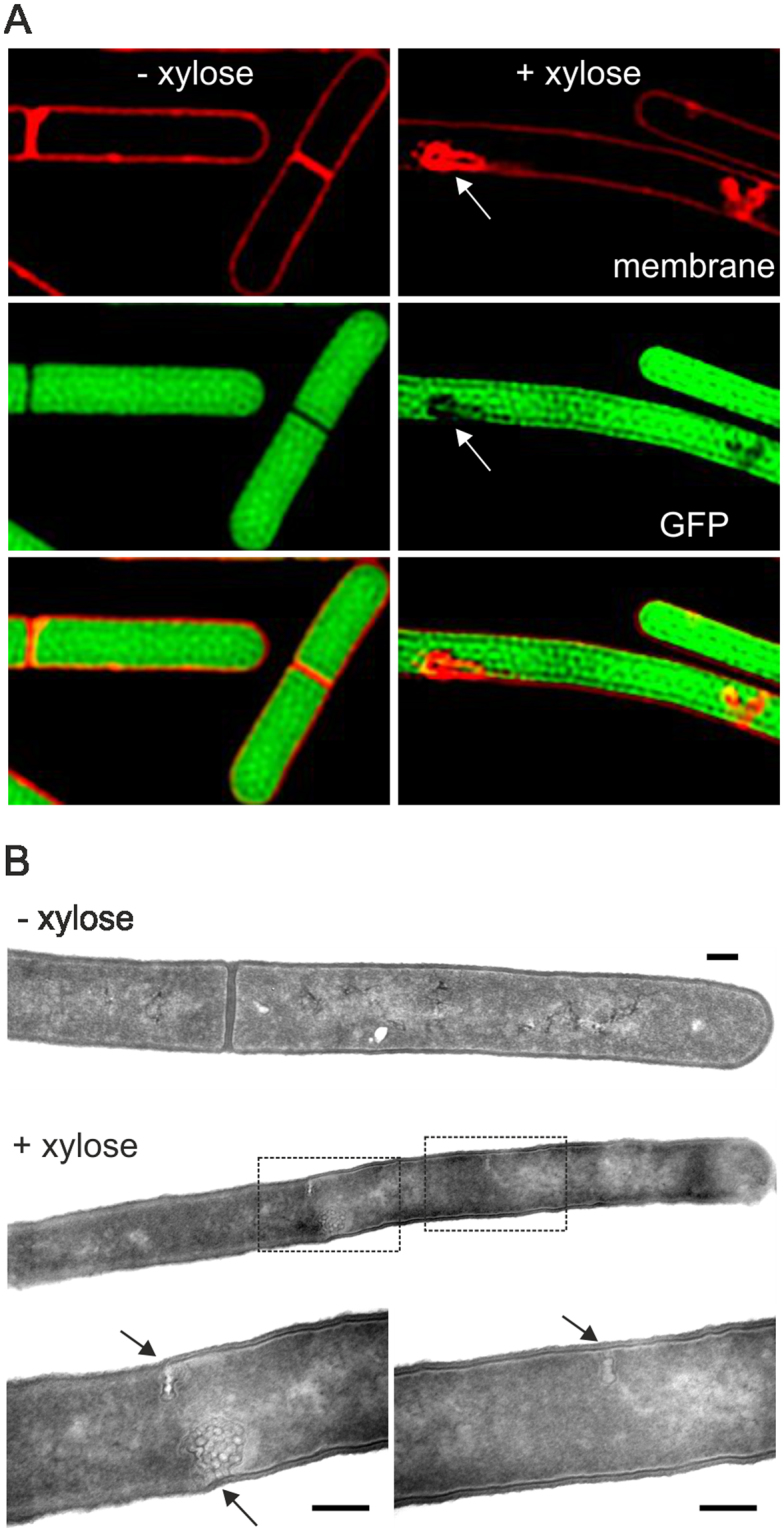
SepF overexpression results in membrane invagination. (**A**) N-SIM microscopy images of strain GYQ257 (*amyE::*P*xyl-sepF aprE::*P*rpsD-gfp*) grown without or with 1% xylose for 3 h to induce SepF expression. Membrane was stained with Nile Red. Membrane invaginations indicated by arrows. (**B**) Transmission electron microscopy images of strain GYQ215 (*amyE::*P*xyl-sepF*) grown without or with 1% xylose. Only longitudinally cut cells revealing whole cells were evaluated. Enlarged images are shown in the lower panels. Arrows indicate membrane invaginations. Scale bar is 200 nm. More examples of these membrane invaginations are shown in Fig. S3.

Despite the filamentous phenotype, cytosolic separation still seems to occur according to the fluorescent membrane stain (Fig. 1B, white arrows). To gain a better view of these apparent cell division sites, we resorted to Structured Illumination Microscopy (SIM) to increase the resolution (Fig. 2A). At higher resolution, these cell division sites appeared to constitute large membrane invaginations, but no clear cell dividing membranes. The absence of cytoplasmic GFP from these areas indicates that these are not SIM artefacts that can originate from the strong fluorescence of membrane probes^25^. Finally, transmission electron microscopy also revealed large membrane invagination in all (>20) cells analyzed (Figs 2B, S3). These large membrane structures are reminiscent of the extra membrane material that is formed when the carboxyltransferase AccDA is overproduced (Fig. S4A). AccDA is involved in the first step of fatty acid synthesis^26^, and its upregulation results in enhanced fatty acid levels and extra membrane synthesis^27^. To test whether the expression of *accDA* was increased due to SepF overexpression, we fused the promoter of *accDA* to the *lacZ* expression reporter. However, β-galactosidase activity measurements did not reveal any significant *lacZ* expression differences when SepF was overexpressed (Fig. S4B).

### Localization of cell division proteins

**Figure 3:**
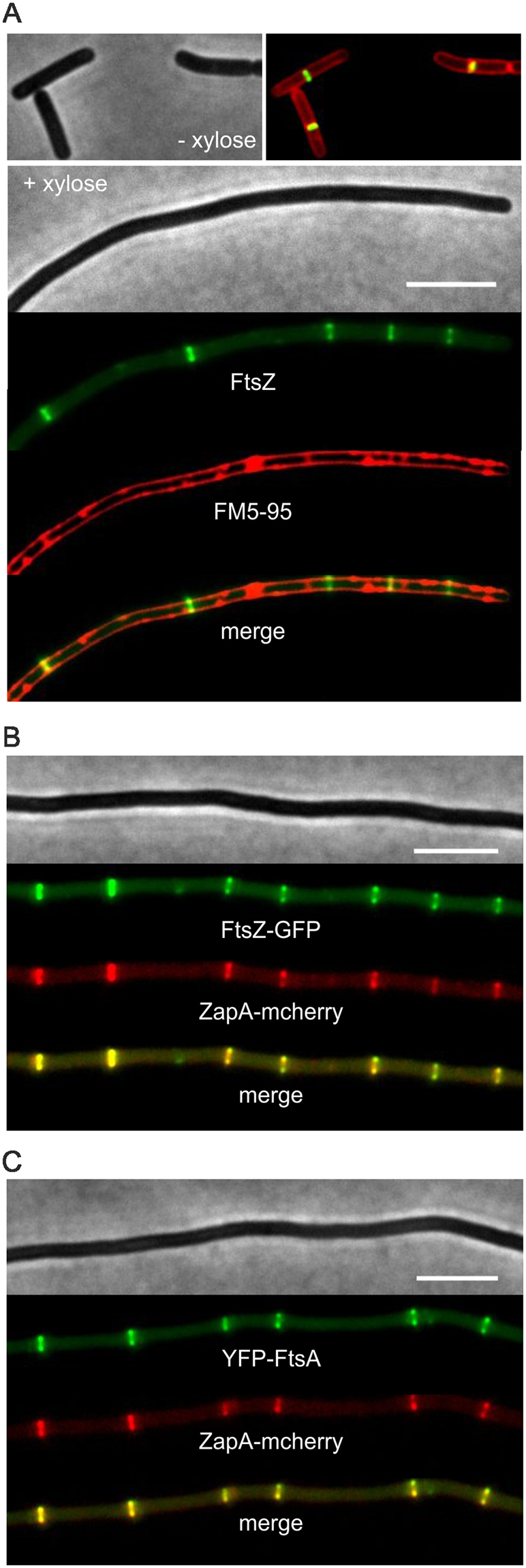
Effect of SepF overexpression on FtsZ, EzrA, ZapA and FtsA localization. Fluorescence light microscopy images of (**A**) strain GYQ298 (*amyE::*P*xyl-sepF ftsZ::ftsZ-gfp*) expressing Ftsz-GFP, (**B**) **s**train GYQ30 (*amyE::*P*xyl*-*sepF ezrA::ezrA-gfp*) expressing EzrA-GFP, and (**C**) strain GYQ212 (*amyE::*P*xyl*-*sepF aprE::Pspac-yfp-ftsA zapA:Pxyl-mcherry-zapA*) expressing YFP-FtsA and mCherry-ZapA, grown without or with 1% xylose to induce SepF expression. Membranes were stained with FM5-95. Scale bar is 5 µm.

To examine whether Z-ring formation is affected by these membrane invaginations, a fluorescent FtsZ-GFP reporter fusion was introduced into the strain (strain GYQ298). Surprisingly, Z-rings are still being formed, and most of them (~76%) do not co-localize with the large membrane invaginations (Figs 3A, S5).

**Figure 4:**
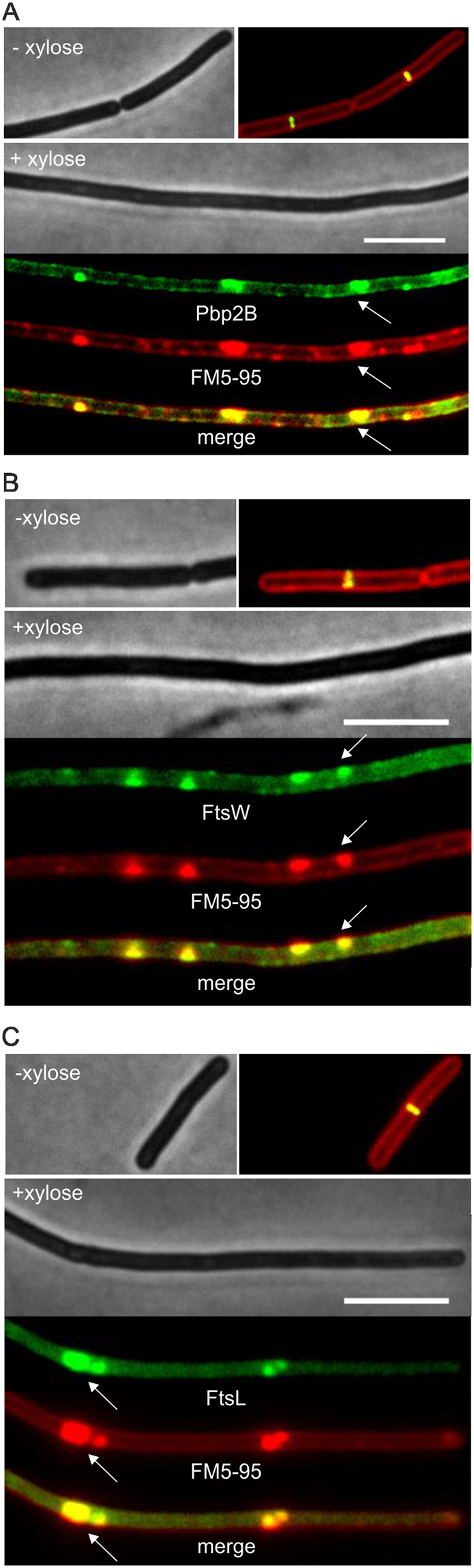
Effect of SepF overexpression on Pbp2B, FtsW and FtsL localization. Microscopic images of (**A**) strain GYQ72 (*amyE::*P*xyl-sepF aprE::*P*spac-gfp-pbpB*) expressing GFP-Pbp2B, (**B**) strain GYQ74 (*amyE::*P*xyl-sepF aprE::*P*spac-gfp-ftsW*) expressing GFP-FtsW, and (**C**) Strain GYQ204 (*amyE::*P*xyl-sepF aprE::*P*spac-gfp-ftsL*) expressing GFP-FtsL, grown in the absence (−) or presence (+) of 1% xylose to induce SepF expression. Membranes were fluorescently stained with FM5-95. Scale bar is 5 µm.

Although overproduction of SepF does not affect Z-ring assembly, there seems to be a problem with Z-ring closure since small Z-rings, indicative of a closing septum, were never observed (~50 cells observed), also not when localization of the early cell division proteins EzrA, FtsA and ZapA were monitored (Fig. 3B,C). This raised the question whether there was a problem with the assembly of the late cell division proteins. When the localization of the transpeptidase Pbp2B was followed using a GFP-Pbp2B reporter fusion, it appeared that overexpression of SepF completely delocalized this protein (Fig. 4A). Strongly fluorescent Pbp2B foci are observed that colocalizes with membrane invaginations (arrow, Fig. 4A). The correlation between the GFP intensity and the membrane dye (FM5-95) intensity indicated that the strongly fluorescent Ppb2B foci are caused by extra cell membrane material (Fig. S6). Importantly, no fluorescent ring-like structures can be seen. The same results were found when the localization of the late division proteins FtsW and FtsL were tracked using fluorescently labelled reporter fusions (Fig. 4B,C). Thus, overproduction of SepF does not impair Z-ring assembly but interferes with the recruitment of late cell division proteins.

### Suppressor mutagenesis

**Figure 5:**
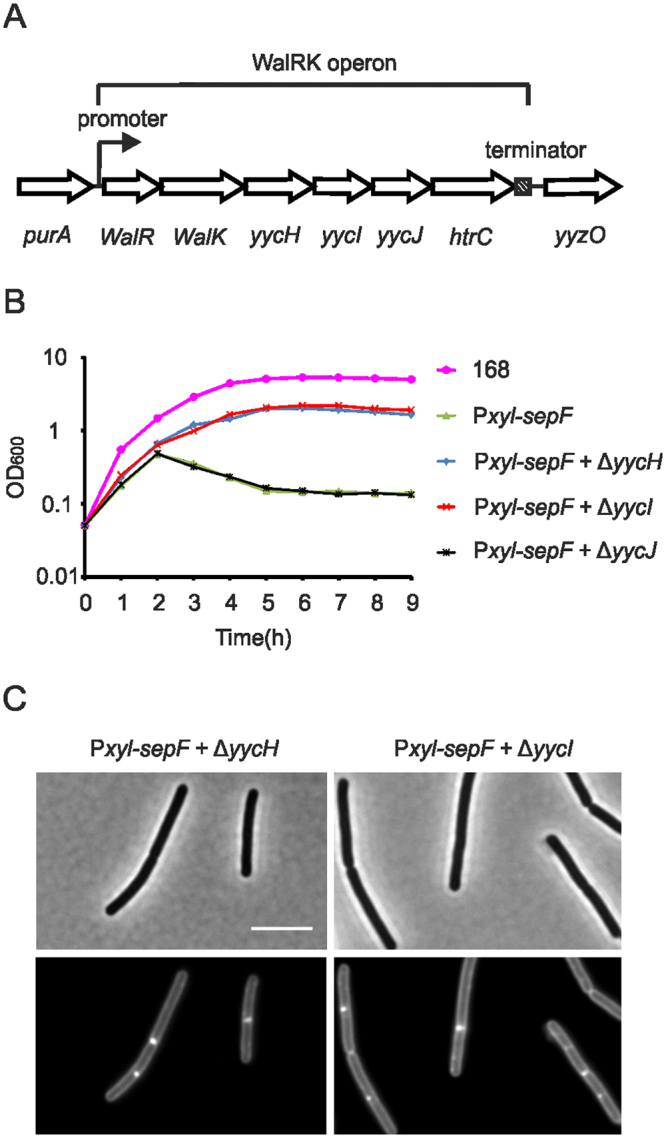
Deletion of *yycH* or *yycI* rescues growth and cell division. (**A**) Map of the WalRK locus. (**B**) Growth curve of strains 168 (wild type), GYQ215 (*amyE::*P*xyl-sepF*), GYQ17 (*amyE::*P*xyl-sepF* ∆*yycH*), GYQ67 (*amyE::*P*xyl-sepF* ∆*yycI*) and GYQ471 (*amyE::*P*xyl-sepF* ∆*yycJ*) grown without or with 1% xylose to induce SepF expression. (C) Phase contrast images of GYQ17 (*amyE::*P*xyl-sepF* ∆*yycH*) and GYQ67 (*amyE::*P*xyl-sepF* ∆*yycI*) cells sampled from the cultures in (**A**) at t = 3 h. Cells were stained with membrane dye FM5-95. Scale bar is 5 µm.

A possible explanation for the failure to form a complete cell division complex could be that high levels of SepF activate a negative regulator that controls assembly of the late cell division proteins. It is important to emphasize that the assembly process of late cell division proteins is not well understood. In fact, after completion of the Z-ring, it takes quite some time, up to 20% of the cell cycle, before the late cell division proteins assemble^28,29^. It is unknown what is responsible for this delay. To identify a possible assembly regulator, we employed transposon suppressor mutagenesis screening. To this end, plasmid pMarB, carrying the mariner transposon TmYLB-1, was transformed into the SepF overproducing strain GYQ215, and approximately 70,000 transposon mutants were screened on xylose containing plates. After backcrossing of several potential suppressor clones, two stable suppressor mutants were isolated that rescued growth when SepF was overproduced. Sequencing of the insertion sites showed that one mutant harbored a transposon insertion into *Pxyl-sepF*, and the other transposon mutant contained an insertion into the gene *yycH* (Fig. 5A).

YycH is a transmembrane protein that negatively regulates phosphorylation of the essential WalRK two component system required for cell wall homeostasis and cell division^30,31^. The response regulator WalR activates the expression of several essential enzymes involved in cell wall remodeling, cell division and cell separation^31,32^. Activated WalR also induces transcription of *ftsZ* ^33^. WalR is phosphorylated by the transmembrane sensor histidine kinase WalK, which is recruited to cell division sites^34^. To confirm that inhibition of *yycH* confers resistance to high SepF levels, a SepF-overproducing strain was constructed in which the complete *yycH* gene was replaced by an erythromycin resistance marker. Indeed, the resulting strain (strain GYQ17) grew normally when 1% xylose was present in the medium and large membrane invaginations were no longer observed (Fig. 5B,C, ~100 cells observed).

**Figure 6:**
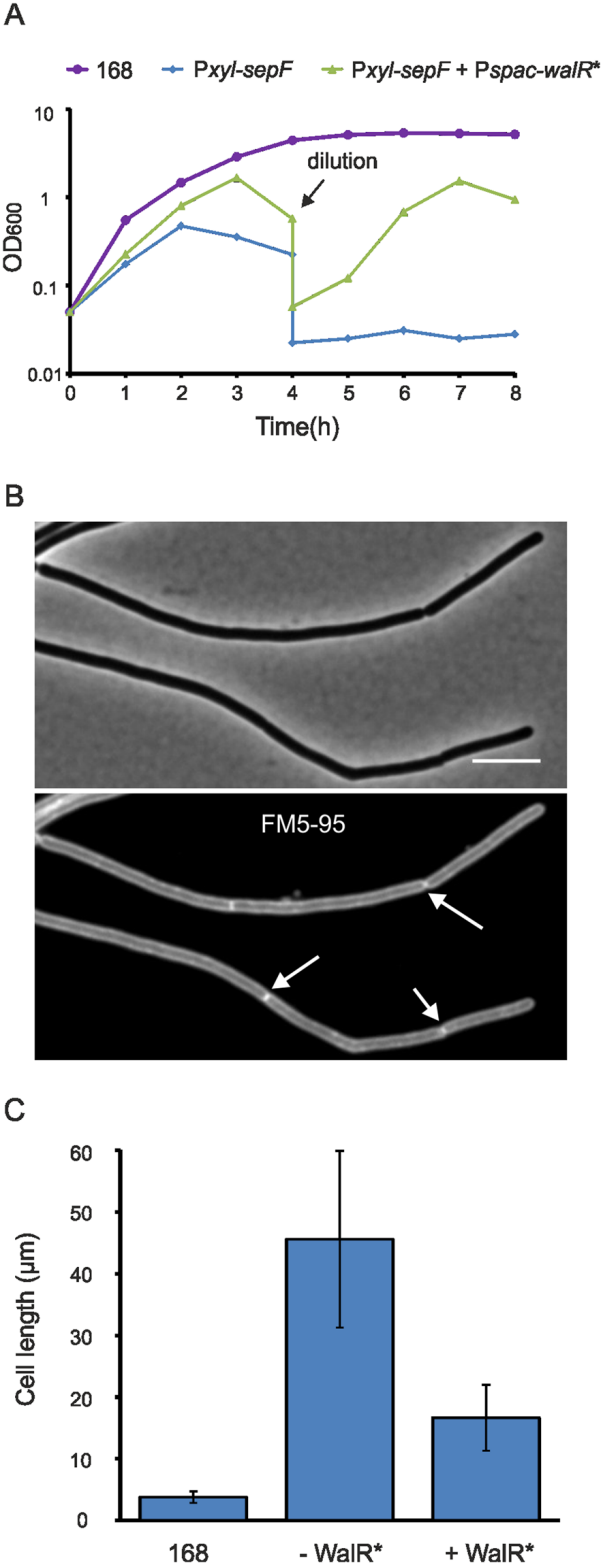
Expression of constitutively activate WalR-R204C. (**A**) Growth of strain 168, GYQ215 (*amyE::*P*xyl-sepF*) and strain GYQ159 (*amyE::*P*xyl-sepF aprE::*P*spac-walR-R204C*) in medium with 1% xylose to induce SepF. In case of strain GYQ159, 1 mM IPTG was added to induce the constitutively active WalR variant R204C (walR*). After 4 h, the culture was diluted. (**B**) Microscopy images of GYQ159 at t = 6 h. Septa are indicated by arrows. Cells were stained with the membrane dye FM5-95. Scale bar is 5 µm. (**C**) Cell length measurement of 168, GYQ215 (−WalR*) and GYQ159 (+WalR*). Samples were taken at t = 3 h.

*YycH* is the third gene in the *walRK* operon (Fig. 5A). The downstream located gene, *yycI*, codes for another transmembrane protein that interacts with and suppresses WalK^35^. Interestingly, replacement of the *yycI* ORF by an erythromycin resistance marker (strain GYQ67) also restored growth (Fig. 5B), while insertion of an erythromycin resistance marker into the downstream located *yycJ* ORF failed to rescue the growth defect caused by SepF overproduction (not shown). These data might suggest that SepF overproduction reduces the activity of WalR. In line with this, when we introduced a constitutively active WalR variant containing the R204C mutation^36^ into the SepF overexpression strain (GYQ159), growth was restored and cells divided again, although not with the same frequency compared to wild type cells as the increase in cell lengths indicated (Fig. 6C).

### WalK delocalization

**Figure 7:**
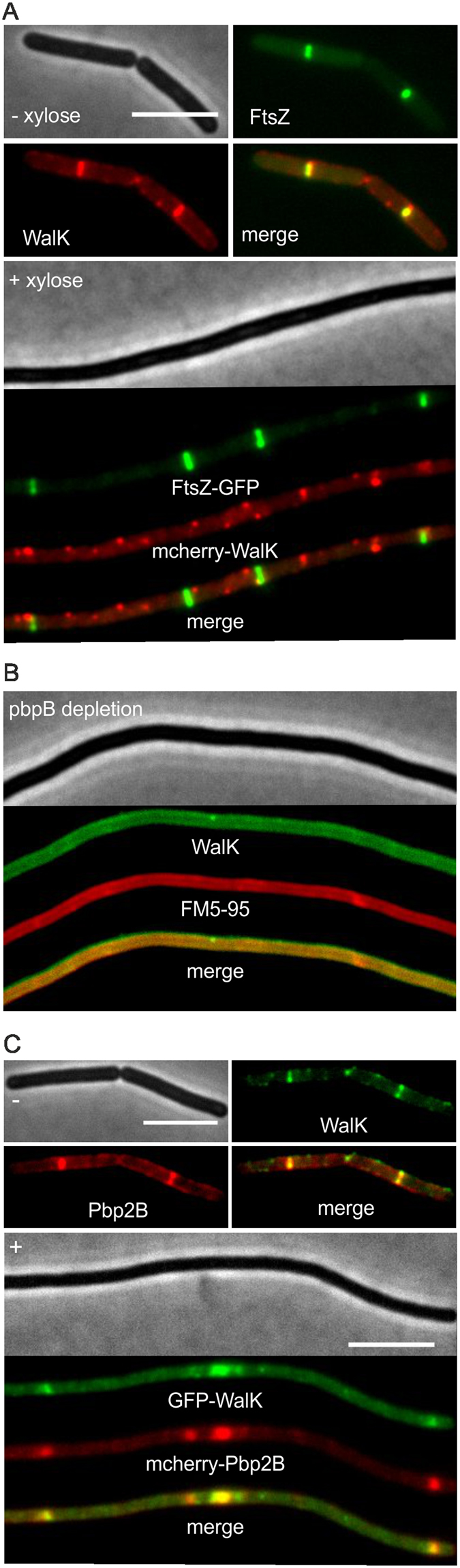
SepF overexpression and *pbpB* depletion delocalize WalK. (**A**) Microscopic images of strain GYQ571 (*amyE::*P*xyl-sepF ftsZ-gfp aprE::*P*spac-mcherry-walK*) expressing both FtsZ-GFP and mCherry-WalK, grown without or with 1% xylose to induce SepF overexpression. Additional images shown in Fig. S7. (**B**) Septum-localization of WalK is abolished in cells depted for *pbpB* (strain GYQ174, *pbpB::*P*spac-pbpB amyE::*P*xyl-gfp-walK*). Membranes were stained with FM5-95. Scale bar is 5 µm.

WalK is recruited to the Z-ring^37^. Possibly, overproduction of SepF interferes with the localization of WalK. To examine this, we introduced a Mcherry-WalK reporter fusion into a SepF overproducing strain that also contained a FtsZ-GFP reporter fusion. Interestingly, the Mcherry-WalK fusion completely delocalized from Z-rings when SepF was overexpressed (Fig. 7A, >50 cells observed).

In a previous study it was shown that the septal localization of WalK requires FtsZ but does not depend on the presence of late cell division proteins^37^. This finding seems to contradict our results since Z-rings are still being formed when SepF is overproduced, yet WalK is delocalized. To confirm that localization of WalK depends on the presence of late cell division proteins, strain GYQ174 was constructed containing the GFP-WalK reporter fusion and an IPTG-inducible *pbpB* gene, coding for the late cell division protein Pbp2B. In *B. subtilis*, assembly of the late division proteins is highly cooperative, and depletion of Pbp2B prevents recruitment of other late cell division proteins to the Z-ring^9^. As shown in Fig. 7B, depletion of Pbp2B also results in complete delocalization of WalK. Since depletion of Pbp2B does not affect Z-ring formation^9^, our data suggest that WalK requires late cell division proteins for septal localization. Therefore, delocalization of WalK due to overexpression of SepF might be a consequence of the failed assembly of late cell division proteins.

### Transcriptome analysis of SepF overexpression

To confirm that SepF overproduction leads to WalR repression, we determined the genome-wide expression profile of SepF overexpressing cells. To this end, wild type strain 168 and a SepF overproduction strain (YK240) where grown in LB to an OD_600_ of ~0.5 (log-phase) in the presence of 1.5% xylose, after which cells were harvested and mRNA isolated for transcriptome analysis. Table 1 lists 64 genes that showed a more than 4-fold expression difference between both strains, with an adjusted p-value of less than 0.05 (reliability measure). To verify the transcriptome data, *lacZ* reporter fusions were constructed with the promoter of the upregulated *ydcFG* and *srfAA* operons, and the down-regulated *mtnKA* operon. The expression response of these fusions were in line with the transcriptome data (Fig. S8). Surprisingly, none of the known WalR-activated genes (e.g. *ftsA*, *ftsZ*, *tagA-F*, Table S1) appeared to be affected by SepF overproduction, and even more puzzling, all the known WalR-repressed genes (*iseA*, *pdaC*, *wapA*, *wapI*) were down-regulated (Table 1, Table S1). Apparently, the cell division defect caused by SepF overproduction does not seem to originate from the repression of WalR.

**Table 1:** Transcriptome analysis of SepF overexpression.

gene	YK240/wt	p.val	function
*ydcF*	65	0.000	hypothetical protein
*ydcG*	37	0.000	hypothetical protein
*ydcH*	36	0.000	hypothetical protein
***sepF***	11	0.000	cell division protein SepF
*bmrC*	7	0.000	multidrug ABC transporter
*bmrD*	6	0.000	multidrug ABC transporter
*catD*	5	0.016	viability in the presence of catechol
*catE*	5	0.016	essential for the viability in the presence of catechol
*srfAC*	4	0.016	surfactin synthesis
*srfAD*	4	0.012	surfactin synthesis
*ycxA*	4	0.001	hypothetical protein
*mtnK*	−90	0.000	methionine salvage
*mtnA*	−70	0.000	methionine salvage
gsiB*	−16	0.023	general stress protein, response to water deficits
gspA*	−12	0.031	general stress protein
yflT*	−10	0.023	general stress protein, ethanol stress
ydaC*	−10	0.007	hypothetical protein
ydaD*	−10	0.029	general stress protein, similar to alcohol dehydrogenase
*wapA*	−10	0.000	contact-dependent growth inhibition protein
*wapI*	−10	0.000	immunity protein against toxic activity of WapA
*yxzC*	−10	0.000	hypothetical protein
*yxiF*	−9	0.000	hypothetical protein
rapB*	−9	0.035	response regulator, control of sporulation initiation
csbC*	−9	0.037	protection against paraquat stress
csbD*	−9	0.023	general stress protein, salt and low temperature stress
*mtnU*	−9	0.016	methionine salvage
ywzA*	−9	0.040	general stress protein
ydaE*	−9	0.023	general stress protein, ethanol and low temperature stress
yhxD*	−9	0.022	general stress protein, salt and ethanol stress
yxbG*	−8	0.016	general stress protein, similar to glucose 1-dehydrogenase
*yxiG*	−8	0.000	hypothetical protein
*yxiH*	−8	0.000	hypothetical protein
*yxiJ*	−8	0.000	hypothetical protein
ysnF*	−8	0.007	general stress protein, ethanol stress
ygxB*	−8	0.021	general stress protein
ydaS*	−8	0.020	hypothetical protein
ytaB*	−8	0.023	general stress protein, salt and ethanol stress
katE*	−8	0.043	general stress protein, catalase
*yxzG*	−7	0.000	hypothetical protein
*yxzI*	−7	0.000	hypothetical protein
*yxiI*	−7	0.000	hypothetical protein
*yxiK*	−7	0.000	hypothetical protein
*yxiM*	−7	0.000	similar to rhamnogalacturonan acetylesterase
yjgD*	−7	0.046	general stress protein, ethanol and paraquat stress
cypC*	−7	0.039	protection against paraquat stress
ywtG*	−7	0.015	general stress protein, similar to metabolite transport protein
yybO*	−7	0.023	similar to permease
chaA*	−6	0.034	calcium export via proton antiporter
ywiE*	−6	0.036	cardiolipin synthesis, protection against paraquat stress
ycbP*	−6	0.023	general stress protein
yqhB*	−6	0.016	general stress protein, protection against oxidative stress
ydaT*	−6	0.023	general stress protein, ethanol and low temperature stress
bmrU*	−6	0.023	general stress protein, multidrug resistance protein
ohrB*	−6	0.036	general stress protein, organic peroxide resistance
katX*	−6	0.032	general stress protein, catalase
yerD*	−6	0.024	general stress protein, protection against paraquat stress
corA*	−6	0.021	general stress protein, similar to magnesium transporter
yfkM*	−5	0.038	general stress protein, detoxification of methylglyoxal
ykgA*	−5	0.006	general stress protein, salt and ethanol stress
yvbG*	−5	0.020	hypothetical protein
ydaG*	−4	0.021	general stress protein, protection against paraquat stress
ydaP*	−4	0.029	general stress protein, ethanol stress
yfkD*	−4	0.018	hypothetical protein
yxzF*	−4	0.013	general stress protein
ycdF*	−4	0.047	general stress protein, ethanol and low temperature stress

Genes are listed based on expression fold difference and selected for >4-fold expression difference between wild type (wt) and strain YK240 (P*xyl-sepF*) grown in the presence of 1.5% xylose to induce SepF. Genes with adjusted P-value (p.val) larger than 0.05 were discarded. Genes marked with ‘*’ belong to the SigB regulon.

Almost ¾ of the 54 down-regulated genes belong to the SigB regulon. Normally expression of the general stress sigma factor SigB is triggered by a wide variety of intra- and extracellular stresses among which membrane integrity affecting substances such as ethanol^38^. Why the production of large membrane invaginations by SepF would reduce rather than induce the SigB stress response is unclear.

### FtsZ and membrane interaction

**Figure 8:**
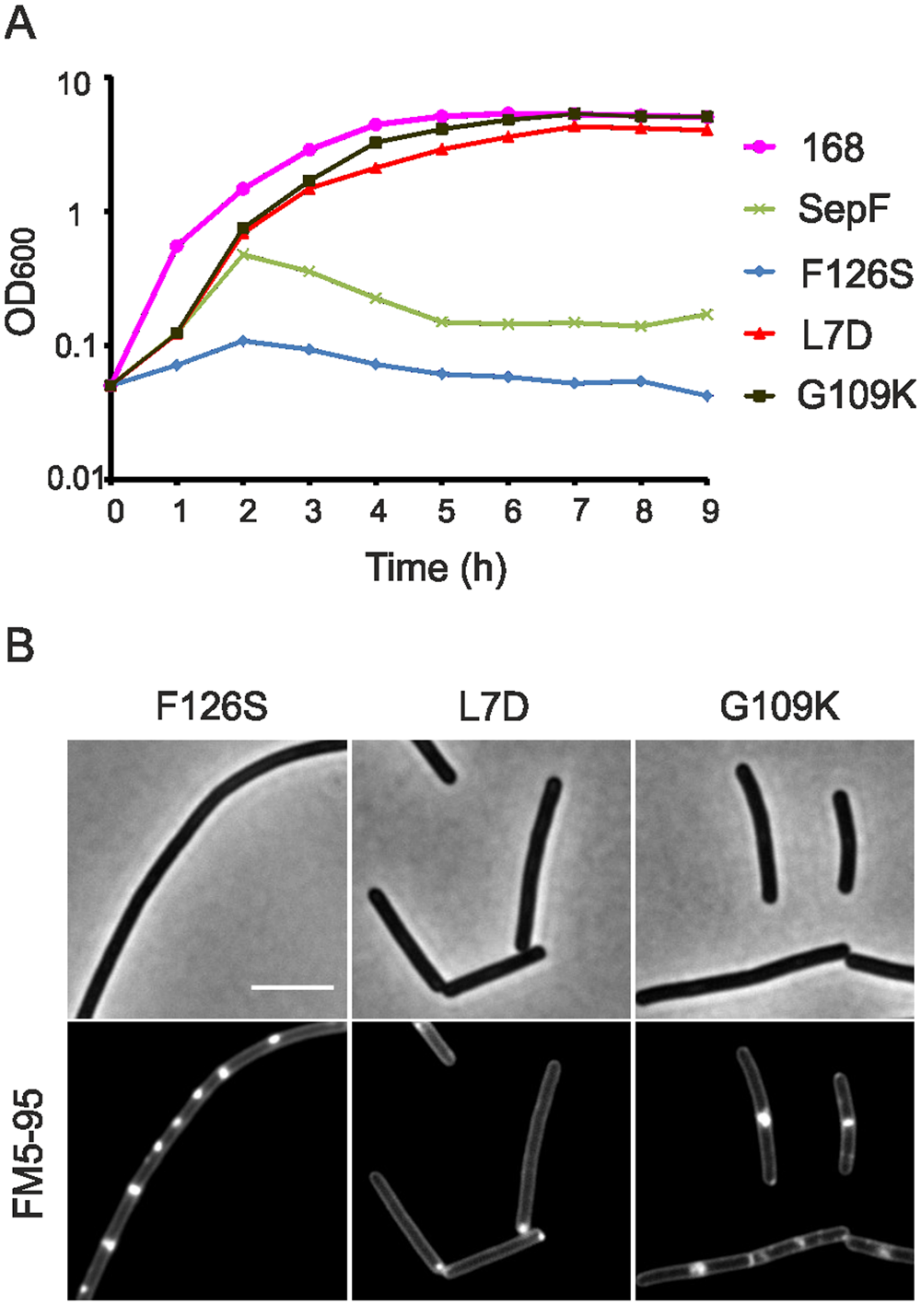
Importance of FtsZ interaction, membrane binding and multimerization domains of SepF. (**A**) Growth of ∆*sepF* strains containing ectopic copies of SepF mutants F126S, L7D or G109K, which either impair FtsZ binding, membrane binding or multimerization, respectively (strain GYQ215 *amyE::*P*xyl-sepF*, strain GYQ207 *amyE::*P*xyl-sepF-F126S* ∆*sepF, strain* GYQ205 *amyE::*P*xyl-sepF-L7D* ∆*sepF*, strain GYQ206 *amyE::*P*xyl-sepF-G109K* ∆*sepF*). Cells were grown in medium supplemented with 1% xylose to induce the SepF mutants. (**B**) Microscopic images of strains GYQ205, GYQ206 and GYQ207 after 3 h growth in the presence of xylose. Membrane invaginations are most apparent when SepF-F126S is expressed. Cells were stained with membrane dye FM5-95. Scale bar is 5 µm.

The transcriptome data did not show a clear down regulation of cell division genes and Z-rings were still formed when SepF was overexpressed. This raised the question whether binding of SepF to FtsZ is actually important for the observed cell division defects. To test this, phenylalanine 126 of SepF was mutated to a serine. It has been shown that this mutation impairs interaction with FtsZ but neither interferes with SepF polymerization (ring formation) nor with membrane binding^5^. Surprisingly, when SepF-F126S was overproduced in a background strain lacking wild type *sepF*, the inhibiting effective on growth was even more severe (Fig. 8A), and the filamentous cells still showed clear membrane invaginations (Fig. 8B). Apparently, SepF does not cause cell filamentation while interacting with FtsZ, which explains why Z-rings are not affected when SepF is overproduced.

This finding raised the question whether the lethal effect of too much SepF depends on its interaction with the cell membrane. To test this, a L7 to D7 substitution was made in the N-terminal amphipathic helix of SepF. This mutation has been shown to impair membrane interaction^5^. When SepF-L7D was overexpressed neither growth nor division was affected (Fig. 8). Induction of SepF containing a G109K mutation, which has been shown to impair polymerization^5^, also had no effect on growth and division, indicating that polymerization of SepF is required for the cell division defect, as well.

### Competition for FtsZ

**Figure 9:**
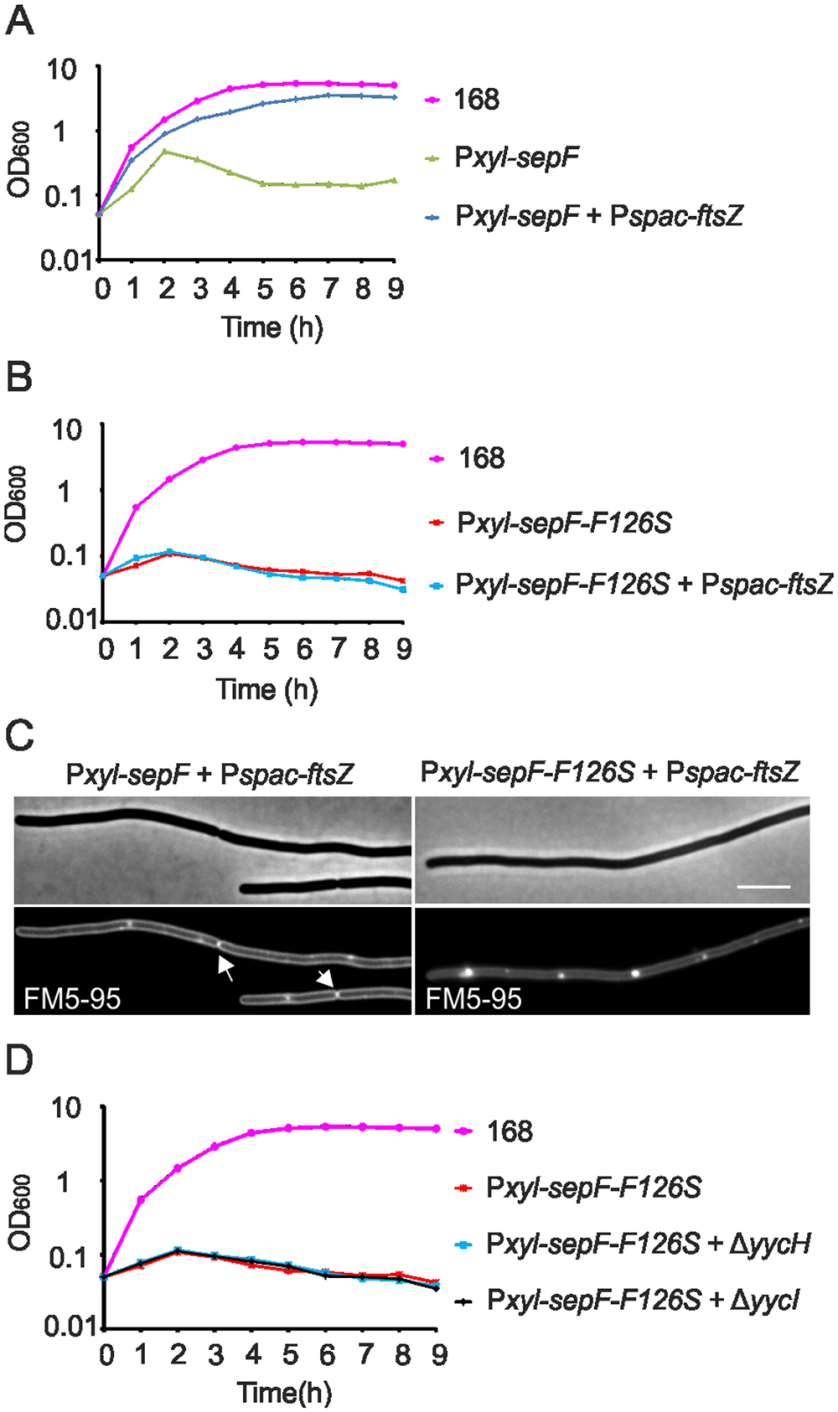
FtsZ induction suppresses division defect. (**A**) Growth curves of strain 168 (wild type), strain GYQ215 (*amyE*::P*xyl-sepF*), and strain GYQ77 (*amyE::*P*xyl-sepF aprE::*P*spac-ftsZ*) in medium containing 1% xylose to induce SepF and 5 mM IPTG to induce FtsZ expression. (**B**) Growth curves of strain 168 (wild type), strain GYQ207 (*amyE*::P*xyl-sepF-F126S* ∆*sepF*) and strain GYQ210 (*amyE*::P*xyl-sepF-F126S* ∆*sepF aprE::*P*spac-ftsZ*) in medium containing 1% xylose to induce SepF-F126S and 5 mM IPTG to induce FtsZ expression. (**C**) Phase contrast and membrane stain of cells from culture GYQ77 (Fig. 9A), and culture GYQ210 (Fig. 9B). Cells were stained with the membrane dye FM5-95. Scale bar is 5 µm. Normal septa are indicated by arrows. (**D**) Growth curves of strain 168 (wild type), strain GYQ185 (*amyE*::P*xyl-sepF-F126S* ∆*sepF*), strain GYQ223 (*amyE*::P*xyl-sepF-F126S* ∆*sepF* ∆*yycH*) and strain GYQ224 (*amyE*::P*xyl-sepF-F126S* ∆*sepF* ∆*yycI*) in medium containing 1% xylose to induce SepF and SepF-F126S.

Overproduction of SepF mutant F126S appeared to be more toxic than overproduction of wild type SepF (Fig. 8A). Possibly, SepF can only exert its inhibiting effect when the protein is not engaged in Z-ring formation. To test this, an extra IPTG-inducible copy of *ftsZ* (*Pspac-ftsZ*) was introduced into the SepF overproduction strain (strain GYQ77). As shown in Fig. 9A, increasing FtsZ levels completely mitigated the growth defect. In line with this finding, when FtsZ levels were increased in cells overexpressing the FtsZ-binding mutant SepF-F126S, growth and cell division were still impaired (Fig. 9B,C).

It appeared that SepF can only perform its cell division inhibiting activity when it is not interacting with FtsZ. Interestingly, it has been shown that overproduction of SepF in a *ftsA* mutant is not lethal (Fig. S9)^39^. Both FtsA and SepF interact with the C-terminal tail of FtsZ^4,40^, suggesting that in a *ftsA* mutant strain more FtsZ is available for binding to SepF. EzrA too binds to the flexible C-terminal domain of FtsZ, and introduction of an *ezrA* deletion into the SepF overexpression strain restored growth and cell division as well (Fig. S9). Apparently, SepF competes with both EzrA and FtsA for binding to the C-terminal domain of FtsZ.

The finding that binding to FtsZ blocks the lethal effect of excess SepF provided an explanation as to why deletion of either *yycH* or *yycI* restored growth when SepF was overexpressed. The absence of these WalK repressors increases WalR phosphorylation, resulting in increased expression of *ftsZ* ^33^. If this is true then a ∆*yycH* or ∆*yycI* mutation should not be able to restore the lethal effect of the SepF-F126S mutant (impaired in FtsZ binding). Indeed, overexpression of SepF-F126S was still lethal when either *yycH* or *yycI* were absent (Fig. 9D).

## Discussion

Overproduction of SepF in the actinomycete *M. smegmatis* and the firmicute *B. subtilis* blocks cell division and results in filamentous cells. Here we provide evidence that this is caused by membrane-associated SepF polymers that are not interacting with FtsZ molecules. These free SepF polymers block recruitment of the late cell division proteins to the Z-ring.

Not much is known about the recruitment of these proteins to the Z-ring, but it is assumed that there is no direct contact between FtsZ and late cell division proteins. In *E. coli* the protein FtsK links early and late division proteins^41,42^, and FtsA interacts with the late cell division proteins FtsN and FtsI^43,44^. *B. subtilis* has two FtsK homologues, SpoIIIE and SftA, but none of these proteins is required for cell division^45,46^. Gram-positive bacteria do not contain an FtsN homologue, and so far there is no clear evidence for a physical interaction between FtsA and late cell division proteins in Gram-positive bacteria. In fact, actinomycetes do not contain a FtsA homologue. A possible explanation for the observed cell division defect is that SepF interacts with one of the late cell division proteins and that overexpression of SepF sterically hinders the formation of a functional link between FtsZ and the late cell division protein.

Alternatively, the large membrane invaginations formed when SepF is overexpressed might hamper the cooperative assembly of late cell division proteins. These membrane invaginations are reminiscent of the excess membrane structures synthesized when the fatty acid synthase subunits AccDA are overexpressed^27^. However, the *accDA* promoter is not activated by SepF overproduction. Also the transcriptome data did not reveal a clear upregulation of genes involved in fatty acid synthesis (Table S2). Presumably, the generation of membrane invagination is related to the mechanism by which SepF binds to the cell membrane. Purified SepF has been shown to strongly deform liposomes and even to cause membrane fusion *in vitro* ^5^. The N-terminus of SepF contains a 10 amino acid long amphipathic helix that inserts into the inner phospholipid layer of the cell membrane. Insertion of such bulky peptide moiety will push tightly packed lipid molecules apart, resulting in substantial bending of the membrane^47^. This effect is enhanced when SepF forms polymers (rings). Therefore, insertion of SepF will curve the membrane towards the cytoplasm and at high concentrations this will lead to invagination of the cell membrane. Possibly, these membrane invaginations, which do not colocalizes with Z-rings (Figs 3, S5), lower the critical concentration of late cell division proteins necessary for the cooperative assembly at Z-rings.

Our transposon experiment suggested that SepF overproduction impairs phosphorylation of WalK, however, this was refuted by the transcriptome data. It turned out that the transposon mutant in *yycH* very likely suppresses the growth defect by increasing the WalKR activity and *ftsZ* expression, thus lowering the concentration of free SepF. Nevertheless, we also found that SepF overproduction prevents binding of WalK to the Z-ring. The likely reason for this is that WalK recruitment relies on the assembly of late cell division proteins. This finding contradicts an earlier study in which it was shown that accumulation of WalK at division sites does not depend on the presence of late proteins^37^. However, in that same study a bacterial two-hybrid assay was used to show that WalK interacts with the late cell division protiens FtsL, DivIB, Pbp2B and FtsW, and in this assay no interaction between WalK and FtsZ could be observed^37^. These bacterial two-hybrid data seems to be more in line with our finding that WalK localization depends on the presence of the late division proteins.

FtsZ binds several proteins during Z-ring formation, four in the case of *B. subtilis*: ZapA, EzrA, FtsA and SepF. The latter three proteins all bind to the flexible C-terminus of FtsZ^4,40,48^. Thus far, it is unknown whether these proteins compete for binding, or whether there is enough space in this ~20 amino acid long domain to simultaneously bind all three proteins. The fact that removing either FtsA or EzrA suppresses the cell division defect of SepF overexpression, as does the induction of FtsZ, suggests that SepF competes with these two proteins for FtsZ binding. Our knowledge of the cell division protein complex, also referred to as the divisome, is still rather limited and it is unclear whether such competition could play a role in the recruitment of late cell division proteins. It has been shown that overexpression of the early cell division proteins ZipA and FtsA in *E. coli* blocks cell division^7,49,50^. However, in these studies it was not further investigated which stage in the division process was impaired. Other studies showed that overexpression of EzrA in *B. subtilis* prevents the formation of Z-rings^6^, and that overexpression of FtsA in *Streptococcus pneumoniae* stimulates Z-ring formation^51^. Our study is the first to show that an imbalance in early cell division proteins affects specifically the recruitment of late cell division proteins.

## Methods

### Bacterial strains and general methods

Strains used in this study are listed in Table S3. *B. subtilis* and its derivatives were grown in LB medium, supplemented with the appropriate antibiotic at the following concentrations: kanamycin 5 µg/ml, chloramphenicol 5 µg/ml, erythromycin 2 µg/ml, spectinomycin 50 µg/ml, and phleomycin 2 µg/ml. *E. coli* Top10 was used for plasmid construction and propagated in LB containing 100 µg/ml ampicillin. All the above strains were cultivated at 37 °C except for the ones harboring FtsZ-GFP, whose detectable fluorescence is stronger at 30 °C. PCR and *E. coli* transformation were carried out using standard methods, and purification of *B. subtilis* chromosomal DNA has been described by Venema *et al*.^52^. Transformation of competent *B. subtilis* was accomplished based on the method of the optimized two-step starvation procedure^53,54^. Gibson assembly cloning was applied for plasmid construction^55^.

### Plasmids construction

PCR primers are listed in Table S4, and all constructs were sequenced to omit possible mutations originating from the PCR reaction. To obtain plasmid pYQ10 harboring the cassette *Pxyl-gfp-walK* for integration into the *amyE* locus, *walK* was amplified using primers YQ99 and YQ101, and genomic DNA of strain 168 as template. The *amyE*-integration vector part was derived by PCR from pHJS105 using primers EKP22 and YQ100. The two fragments, with 20 bp overlapping sequences, were assembled using Gibson assembly (see New England Biolabs protocol for details). pYQ10 was used as vector template using PCR and primers YQ451 and YQ452, and the mCherry region was derived from genomic DNA of EKB36 using primers YQ450 and YQ453. The two fragments were assembled, resulting in the mCherry-WalK fusion (pYQ141).

GFP reporter fusions integrated into the *aprE* locus were constructed as follows. Firstly, plasmids carrying P*xyl-gfp-pbpB*, P*xyl-gfp-ftsW* and P*xyl-gfp-ftsL* for integration into the *amyE* locus were constructed, whereby, *pbpB*, *ftsW*, and *ftsL* were amplified using genomic DNA and primer pairs EKP31 & EKP32, EKP38 & EKP39, EKP33 & EKP34, respectively. The *amyE*-integration vector part was derived from pHJS105 using primers EKP22 and EKP30. The fragments were assembled using Gibson assembly, resulting in pEKC12, pEKC14 and pEKC13, respectively. Plasmid pTNV9 containing *Erm*-*lacI*-P*spac-gfp* for *aprE* locus integration was assembled using two fragments. The plasmid fragment carrying *erm*-*lacI*-P*spac* and *aprE* flanking sequences was derived from pAPNC213 *erm* using primers TerS135 and TerS136, and the GFP fragment was amplified with primers TerS139 and TerS140, and pHJS105 as template. The *aprE* integration plasmids carrying the GFP reporter fusions *aprE*::P*spac-gfp-ftsW*, *aprE*::P*spac-gfp-ftsL*, *aprE*::P*spac-gfp-pbpB* and *aprE*::P*spac-gfp-walK*, were constructed in the following way. Firstly, the *gfp-ftsW, gfp-ftsL, gfp-pbpB* and *gfp-walK* gene fusions were isolated by PCR using primer pairs YQ41 and YQ42 and template DNA pEKC14, pEKC13, pEKC12 and pYQ10, respectively. The vector part containing the P*spac* promoter and *aprE* flanking sequences was amplified with primers YQ43 and YQ44 and plasmid pTNV9 as template. Subsequently, the GFP reporter fusions and the vector part were ligated using Gibson assembly, resulting in pYQ01, pYQ02, pYQ03 and pYQ11, respectively.

To construct plasmid pYQ14 harboring an IPTG-inducible WalR-R204C mutant, full length *walR* with its own ribosome-binding site was isolated using primers YQ142 and YQ75, and the vector fragment was amplified using primers YQ76 and YQ143 and the *aprE*-integration plasmid pAPNC213 *Kan* as template. The two PCR products were fused using Gibson assembly resulting in plasmid pYQ13. Plasmid pYQ14 was constructed with quick-change mutagenesis of plasmid pYQ13, using primers YQ78 and YQ79.

The *accDA* and *ydcF* promoter-*lacZ* reporter fusions were constructed by amplifying the *accDA* or *ydcF* promoter regions from the chromosome using primer pairs YQ102 & YQ103, and YQ94 & YQ95, respectively. PCR products were digested with BamHI and HindIII, and ligated into digested pMutin4, resulting in plasmids pYQ40 and pYQ05, respectively. pYQ87 the promoter-*lacZ* reporter plasmid for *aprE* locus integration, was assembled from 4 PCR fragments. The plasmid fragment carrying the *aprE* flanking sequences was derived from pTNV9 using primers YQ73 and YQ218. The spectinomycin resistance cassette was amplified using primers YQ72 and YQ219 and pHJS105 as template. The terminator fragment containing three terminators (t1, t2, t0 from *rrnB* operon of *E. coli*) was amplified from pMutin4 with primers YQ220 and YQ216. The *lacZ* reporter fragment was amplified with primers YQ214 and YQ217 using pMutin4 as template. These 4 PCR fragments were ligated with Gibson assembly resulting in pYQ87. The three terminators are positioned upstream of the promoter-*lacZ* reporter and downstream of the spectinomycin cassette. P*srfAA* and P*mtnK* promoter regions were amplified from genomic DNA with primer pairs YQ236 & YQ237, and YQ246 & YQ247, respectively, and assembled with a vector fragment derived from pYQ87 using primers YQ214 and YQ215, resulting in pYQ47 and pYQ56, respectively.

The P*rpsD*-gfp reporter fusion was amplified with primers YQ72 and YQ42, using chromosomal DNA of strain BSS421 as template. The vector containing *aprE* flanking sequences was amplified with YQ43 and YQ73, and pTNV9 as template. The two fragments were assembled resulting in pYQ73. To construct plasmid pTNV60, for replacement of the chloramphenicol resistance marker (*Cm*) with the kanamycin resistance marker (*Kan*) in the *B. subtilis* genome, first plasmid pTNV42 was constructed using pUC19 amplified by primer pair TerS117 and TerS118, in which the *Cm* region was inserted. This Cm cassette was amplified with TerS125 and TerS126, and plasmid pAPNC213 *Cm* as template. Subsequently, pTNV42 was used as vector template in a PCR amplification reaction using primers TerS257 and TerS258. The *Kan* region was derived from pMarB using primers TerS259 and TerS260. Both fragments were assembled, resulting in pTNV60.

### Strain construction

The relevant *B. subtilis* strains are listed in Table S3. GYQ215 was constructed by transforming chromosomal DNA of YK240 to 168. To construct the mutant SepF-L7D controlled by P*xyl* at the *amyE* locus, the upstream region was amplified by PCR using primers YQ53 and YQ150, and the downstream region was amplified with primers YQ149 and YQ52. Chromosomal DNA of strain GYQ215 was used as template for the PCR reactions, and the two PCR fragments were assembled by Gibson assembly and directly transformed to competent 168 cells, resulting in strain GYQ178. The same procedure was followed for the construction of P*xyl-sepF*-G109K and P*xyl-sepF*-F126S. Upstream regions of P*xyl-sepF*-G109K and P*xyl-sepF*-F126S were amplified with primer pairs YQ53 & YQ152, and YQ53 & YQ154, and the downstream regions were amplified using primer pairs YQ52 & YQ151, and YQ52 & YQ153, respectively. The upstream and downstream fragments were ligated with Gibson assembly and transformed to competent 168 cells, generating strain GYQ179 and GYQ180, respectively.

Strain GYQ124 carrying P*xyl-gfp-walK* at the *amyE* locus was constructed by transforming pYQ10 to competent 168 cells. All the integrations at the *amyE* locus were verified using colony PCR with primers TerS350 and TerS351 and by sequencing if necessary. Plasmids pYQ01, pYQ02, pYQ03, pYQ11 and pYQ141 were transformed to competent 168 cells, resulting in strains GYQ81, GYQ203, GYQ73, GYQ144 and GYQ570, respectively. Strains GYQ195, GYQ217 and GYQ254 were constructed by transforming pYQ47, pYQ56 and pYQ73, respectively to competent 168 cells. To construct strain GYQ152 carrying the P*spac-walR*-R204C reporter at the *aprE* locus, pYQ14 was transformed to competent 168 cells. All *aprE* locus integrations were verified by colony PCR with primers TerS352 and TerS353.

Strain GYQ135 harbouring the P*accDA-lacZ* reporter and GYQ132 harbouring the P*ydcF-lacZ* reporter at their native locus were constructed by transforming plasmid pYQ40 and pYQ05, respectively to competent 168 cells followed by single crossover. Integration of reporter P*ydcF-lacZ* was checked by colony PCR using primers YQ105 and YQ98, and integration of reporter P*accDA-lacZ* was verified by colony PCR with primers YQ104 and YQ98. To replace the genomic *Cm* of EKB36 with *Kan*, the plasmid pTNV60 was transformed into competence EKB36 carrying P*xyl-mcherry-zapA*, resulting in GYQ29. To replace the genomic *Cm* of 4057 to *Kan*, the plasmid pTNV60 was transformed into competence 4057 carrying *ezrA-gfp*, resulting in GYQ28.

### Fluorescence light microscopy

Overnight cultures in LB medium were inoculated into fresh LB medium supplemented with the relevant antibiotics and inducers. For the strains harbouring the *pbpB* depletion construct (e.g. strain GYQ174), the overnight culture was washed by centrifugation to remove IPTG, and inoculated into fresh LB without IPTG. For fluorescence microscopy, samples were taken at exponentially growth and mounted onto microscope slides coated with a thin layer of 1.2% agarose. Images were acquired with Nikon CoolSnap camera with a Zeiss Axiovert 200 M epifluorescence microscope running MetaMorph software, or a Nikon N-SIM microscope equipped with a Nikon APO TIRF × 100/1.49 lens in both EPI and 2D-SIM modes with 488 nm solid-state lasers. When required, cells were incubated with membrane dye FM5-95 (90 µg/ml) or MitoTracker green (500 nM) for 5 min prior to immobilization on microscope slides. When membranes were visualized with Nile Red under the SIM microscope, the coverslip was coated with a poly-dopamine film freshly prepared with 2 g/l dopamine solution^56^. For DNA staining, 2 µg/ml DAPI was added to the medium after which cells were prepared for microscopy. For cell length measurement, cells were mixed with membrane dye FM5-95 (90 µg/ml), prior to microscopic examinations, and the ChainTracer, based on ImageJ Plugin ObjesctJ, was used for the measurement of cell length^57^.

### Transposon mutagenesis screen

Random transposon mutagenesis of the strain GYQ215 (*amyE*::P*xyl-sepF*) was performed using the plasmid pMarB carrying mariner transposable element TnYLB-1 as described^58^. The competence GYQ215 cells were transformed with plasmid pMarB and incubated at 30 °C. Several individual colonies carrying pMarB were picked and grown at 30 °C overnight, and then inoculated into fresh LB medium at 30 °C until OD_600_ reached 0.2–0.3, after which growth was continued at 48 °C for 2 h. Aliquots were frozen and stored at −80 °C. Serial dilutions of each culture were spread on LB plates with kanamycin or erythromycin, and incubated at 50 °C overnight to lose the plasmid pMarB. The aliquot with the highest ratio of Kan^R^/erm^R^ colonies was chosen for further experiment. This aliquot was plated on LB plates supplemented with kanamycin and 1% xylose and incubated at 50 °C overnight. Individual colonies that grew with kanamycin and 1% xylose but failed to survive on plate containing erythromycin and 1% xylose, were picked and checked under the microscope for loss of filamentous phenotype. Two rounds of backcrosses were performed as follows. Chromosome DNA of selected colonies was transformed into wild type 168, and chromosome DNA from the resulting transformant was transformed into GYQ215 to confirm the linkage between transposon insertion and loss of filamentous phenotype. Finally, inverse PCR amplification and subsequent sequencing was performed to determine the transposon insertion site.

### Transcriptome analysis

For transcriptome analyses a 8 × 15 k Custom Agilent microarray was used. The NCBI annotation BSU41030 *B. subtilis* 168 genome 2006-05-02 GenBank, containing information for 4105 transcripts, was used to design three probes per transcript. Overnight cultures of wild type strain 168 and strain strain YK240 were diluted (1:100) into fresh LB medium supplemented with 1.5% xylose and grown at 37 °C until OD_600_ ~0.5, after which the cells were collected by centrifugation, flash frozen in liquid nitrogen and stored at −80 °C. Frozen pellets were grounded and subjected to RNA extraction as described previously^59^, yielding RIN values of ≥9.6. Labelling was performed by reverse transcription using random octamers, incorporating Cy3 for the test samples and Cy5 for the common reference, as described^60^. The common reference was a pool of equal amounts of total RNA taken from all test samples. Hybridization, washing, and scanning was performed as described in the Two-Color Microarray-Based Gene Expression Analysis manual (Version 6.6, Agilent Technologies). Briefly, hybridization mixtures were made by combining 300 ng test (Cy3) and 300 ng common reference (Cy5) material and were subsequently hybridized to the Agilent SurePrint Custom 8 × 15 k microarrays G2509F (Agilent Technologies). Three biological replicates were used for each strain. Raw and normalized data from all arrays were subjected to various quality control checks^59^. Normalized expression values were calculated using the robust multi-array average (RMA) algorithm^61^, and collecting and summarizing the intensity values of probes associated with a specific BSU locus tag. Differences in gene expression were statistically analysed using the Limma package in R 2.14.1 (http://cran.r-project.org/). Empirical Bayes test statistics were used for calculating P-values^62^, and for calculating false discovery rate corrected P-values (adjusted P-values)^63^. Gene expression data and array design have been deposited at the public repository Gene Expression Omnibus, accession number. Functionality of selected genes (Table 1) were assigned using Subtiwiki^64^.

Expression of several genes was verified by *lacZ* promoter fusions and β-galactosidase assays. These were performed using exponentially growing cultures according to the method described by Daniel *et al*.^65^.

### Western blotting

Samples of exponentially growing cultures were collected, spun down, and washed twice with TE buffer (100 mM Tris-HCl pH 7.5, 1 mM EDTA) and stored on ice. Cell pellets were resuspended in 500 µl buffer (10 mM Tris-HCl PH 7.5) and disrupted by sonication. Cell debris was removed by centrifugation. Protein concentrations were determined with Bio-Rad protein assay and equal amounts of protein were loaded onto a SDS-PAGE gel. Proteins were transferred onto a Hybond-P PVDE membrane (GE Healthcare) using a wet procedure according to standard Western Blotting protocols. A 1:3000 dilution of rabbit polyclonal anti-SepF was used, and anti-rabbit horseradish peroxidase-linked antiserum as secondary antibody with a dilution of 1:10000. Protein bands were imaged using the ImageQuant LAS 4000 mini digital imaging system (GE Healthcare).

### Transmission electron microscopy

Cells were placed on an agarose patch, allowed to dry for 2 min, and subsequently fixed with 5% glutaraldehyde in 0.1 M cacodylate buffer (pH 7.4) for 20 min. Agarose-embedded samples were washed three times with 0.1 M cacodylate buffer (pH 7.4) and then stained with a 1:1 mixture of osmium tetroxide (1%) and K3[Ru(III)(CN)6] (1%) for 30 min, followed by three times washing with water. Samples were then dehydrated in an incubation series with rising concentrations of ethanol as follows: 5 min 30% ethanol, 5 min 50% ethanol, 15 min 70% ethanol (twice), 60 min 80% ethanol, 15 min 90% ethanol, 15 min 96% ethanol, 15 min 100% ethanol, 30 min 100% ethanol (water-free), 5 min propylene oxide, 30 min 1:1 EPON/propylene oxide, 30 min 2:1 EPON/propylene oxide. Samples were then covered with fresh EPON, incubated over night at room temperature, and subsequently allowed to polymerize at 65 °C for 36 h prior to ultrathin sectioning. Pictures were taken with a Jeol 1010 TEM at an electron voltage of 80 kV.

## Electronic supplementary material

supplementary material
