# Supplementary material for: Free SepF interferes with recruitment of late cell division proteins
Source: Sci Rep. 2017 Dec 5;7:16928. doi: 10.1038/s41598-017-17155-x (PMC5717166; doi:10.1038/s41598-017-17155-x)
Supplement: Supplementary file 1 — supplementary material [file 41598_2017_17155_MOESM1_ESM.pdf]

Supplementary Information

Free SepF interferes with recruitment of late cell division proteins

Yongqiang Gao, Michaela Wenzel, Martijs J. Jonker and Leendert W. Hamoen

Table S1: Effect of SepF overexpressing on WalR regulon

Table S2: Effect of SepF overexpressing on genes involved in fatty acid synthesis

Table S3: Strains and plasmids used in this study

Table S4: Primer sequences used in this study

References

Fig. S1: SepF overexpression

Fig. S2: Aberrant membrane invaginations

Fig. S3: Transmission electron microscopy

Fig. S4: High SepF levels do not induce AccDA expression

Fig. S5: Most Z-rings do not co-localize with membrane invaginations

Fig. S6: Correlation of GFP-Pbp2B and FM5-95 fluorescence signals

Fig. S7: WalK is delocalized from Z-rings

Fig. S8. Effect of SepF overexpression on gene expression

Fig. S9: Suppression of growth and cell division defect by deleting either *ezrA* or *ftsA*

Table S1: Effect of SepF overexpressing on WalR regulon

Genes belonging to the WalR regulon [1-4] are listed with their fold expression difference (YK240/168) and ajusted p-value. “-” represents down-regulation genes.

**Activated genes**			
	**YK240/wt**	**p.val**	**function**
cwlO	1.5	0.169	cell wall synthesis, cell elongation
ftsA	-1.0	0.991	formation of Z-ring
ftsZ	-1.0	0.955	formation of Z-ring
lytE	-2.7	0.004	major autolysin, cell elongation and separation
mreBH	1.7	0.178	cell shape determation
sigI	1.3	0.593	control of a class of heat shock genes
rsgI	1.5	0.364	control of SigI activity
tagA	1.1	0.826	biosynthesis of teichoic acid
tagB	1.3	0.626	biosynthesis of teichoic acid
tagD	1.0	0.996	biosynthesis of teichoic acid
tagE	1.1	0.962	biosynthesis of teichoic acid
tagF	1.0	0.982	biosynthesis of teichoic acid
ydjM	3.0	0.071	may be involved in cell wall metabolism
ykvT	-1.1	0.957	hypothetical protein
yocH	-1.3	0.462	cell wall turnover
**Repressed genes**			
	**YK240/wt**	**p.val**	**function**
iseA	-3.0	0.023	protection against cell envelope stress
pdaC	-2.2	0.073	cell wall modification
wapA	-9.7	0.000	contact-dependent growth inhibition protein
wapI	-10.1	0.000	immunity protein against toxic activity of WapA

Table S2: Effect of SepF overexpressing on genes involved in fatty acid synthesis

Genes belonging to fatty acid synthetic systems were listed with their fold expression variation (YK240/168) and ajusted p-values. “-” represents down-regulated genes.

**Genes involving in fatty acid synthesis**	**YK240/wt**	**p.val**	**function**
accA	-1.02	0.962	acetyl-CoA carboxylase (alpha subunit)
accB	1.01	0.983	acetyl-CoA carboxylase (biotin carboxyl carrier subunit)
accC	1.00	0.995	acetyl-CoA carboxylase (biotin carboxylase subunit)
accD	-1.04	0.889	acetyl-CoA carboxylase (beta subunit)
acpA	-1.08	0.771	acyl carrier protein
acpS	1.14	0.512	acyl-carrier protein synthase, 4-phosphopantetheine transferase
birA	1.11	0.499	regulation of biotin synthesis, addition of biotin to proteins
fabD	1.14	0.746	malonyl CoA-acyl carrier protein transacylase
fabF	1.06	0.892	β-ketoacyl-acyl carrier protein synthase II, involved in the control of membrane fluidity
fabG	1.17	0.628	β-ketoacyl-acyl carrier protein reductase
fabHA	1.06	0.883	β-ketoacyl-acyl carrier protein synthase III, principal condensing enzyme responsible for the initiation of fatty acid synthesis in non-stressed B.subtilis cells
fabHB	1.41	0.517	β-ketoacyl-acyl carrier protein synthase III
fabI	1.11	0.664	enoyl-acyl carrier protein reductase
fabL	-1.01	0.980	enoyl-acyl carrier protein reductase
fapR	1.15	0.742	repressor of fatty acid synthetic genes
plsC	1.09	0.705	acyl-ACP:1-acylglycerolphosphate acyltransferase
plsY	-1.13	0.421	acylphosphate:glycerol-phosphate acyltransferase
plsX	1.18	0.680	acyl-acyl carrier protein (ACP): phosphate acyltransferase, catalyzes the synthesis of the intermediate fatty acyl-phosphate, coordinates membrane synthesis with cell division
ycsD	1.08	0.681	β-hydroxyacyl-ACP dehydratase
ywpD	1.48	0.523	two-component orphan sensor kinase
ywpB	-1.13	0.677	β-hydroxyacyl-ACP dehydratase

Table S3: Strains and plasmids used in this study

All strains were made in the *B. subtilis* 168 wild type background. Antibiotic resistance genes were abbreviated as follows: *kan* (kanamycin), *cat* (chloramphenicol), *erm* (erythromycin), *phleo* (phleomycin), *spec* (spectinomycin), *bla* (ampicillin).

Strains	Relevant features or genotype	Construction or reference
B.Subtilis		
168	trpC2	[5]
YK240	amyE::cat Pxyl-sepF	Yoshi Kawai, unpub.
YK1738	amyE::spec Pxyl-accDA	[6]
GYQ215	amyE::cat Pxyl-sepF	168 transformed with YK240
874	ftsZ::spec ftsZ-gfp	Laboratory stock
GYQ298	amyE::cat Pxyl-sepF ftsZ::spec ftsZ-gfp	GYQ215 transformed with 874
GYQ73	aprE::erm Pspac-gfp-pbpB	This study
GYQ81	aprE::erm Pspac-gfp-ftsW	This study
GYQ203	aprE::erm Pspac-gfp-ftsL	This study
GYQ72	amyE::cat Pxyl-sepF aprE::erm Pspac-gfp-pbpB	GYQ73 transformed with GYQ215
GYQ74	amyE::cat Pxyl-sepF aprE::erm Pspac-gfp-ftsW	GYQ81 transformed with GYQ215
GYQ204	amyE::cat Pxyl-sepF aprE::erm Pspac-gfp-ftsL	GYQ203 transformed with GYQ215
GYQ135	chr: (pMUTIN4 PaccDA-lacZ erm)	This study
GYQ136	chr: (pMUTIN4 PaccDA-lacZ erm) amyE::cat Pxyl-sepF	GYQ135 transformed with GYQ215
TB74	yycH::erm	Laboratory stock
BKE40380	yycI::erm	BGSC stock
BKE40370	yycJ::erm	BGSC stock
GYQ201	yycH::erm	168 transformed with TB74
GYQ202	yycI::erm	168 transformed with BKE40380
GYQ470	yycJ::erm	168 transformed with BKE40370
GYQ17	amyE::cat Pxyl-sepF yycH::erm	GYQ201 transformed with GYQ215
GYQ67	amyE::cat Pxyl-sepF yycI::erm	GYQ202 transformed with GYQ215
GYQ471	amyE::cat Pxyl-sepF yycJ::erm	GYQ470 transformed with GYQ215
bSS421	amyE::spec PrpsD-gfp	Syvertsson, unpub.
GYQ254	aprE::spec PrpsD-gfp	This study
GYQ257	amyE::cat Pxyl-sepF aprE::spec PrpsD-gfp	GYQ254 transformed with GYQ215
GYQ132	chr : (pMUTIN4 PydcF-lacZ erm)	This study
GYQ133	chr : (pMUTIN4 PydcF-lacZ erm) amyE::cat Pxyl-sepF	GYQ132 transformed with GYQ215
GYQ195	aprE::spec PsrfAA-lacZ	This study
GYQ199	amyE::cat Pxyl-sepF aprE::spec PsrfAA-lacZ	GYQ195 transformed with GYQ215
GYQ217	aprE::spec PmtnK-lacZ	This study
GYQ218	amyE::cat Pxyl-sepF aprE::spec PmtnK-lacZ	GYQ217 transformed with GYQ215
GYQ144	aprE::erm Pspac-gfp-walK	This study
GYQ139	amyE::cat Pxyl-sepF aprE::erm Pspac-gfp-walK	GYQ144 transformed with GYQ215
GYQ124	amyE::spec Pxyl-gfp-walK	This study
3294	chr::(Pspac-pbpB Kan) divIVA::PdivIVA-gfp-divIVA cat	[7]
TNVS87	chr:: (Pspac-pbpB Kan)	168 transformed with 3294
GYQ174	chr:: (Pspac-pbpB Kan) amyE::spec Pxyl-gfp-walK	GYQ124 transformed with TNVS87
GYQ570	aprE::erm Pspac-mcherry-walK	This study
GYQ571	amyE::cat Pxyl-sepF ftsZ::spec ftsZ-gfp aprE::erm Pspac-mcherry-walK	GYQ298 transformed with GYQ570
GYQ152	aprE::Kan Pspac-walR* R204C	This study
GYQ159	amyE::cat Pxyl-sepF aprE::Kan Pspac-walR* R204C	GYQ152 transformed with GYQ215

TB07	*ftsA::erm*	Laboratory stock
PG49	*ezrA::spec*	[8]
TNVS158	*ezrA::spec*	168 transformed with PG49
TNVS101	*aprE*::*spec* P*spac-ftsZ*	Saaki, unpub.
GYQ10	*amyE::cat* P*xyl-sepF ftsA::erm*	GYQ215 transformed with TB07
GYQ130	*amyE::cat* P*xyl-sepF ezrA::spec*	GYQ215 transformed with TNVS158
GYQ77	*amyE::cat* P*xyl-sepF aprE*::*spec* P*spac-ftsZ*	TNVS101 transformed with GYQ215
GYQ178	*amyE::cat* P*xyl-sepF-L7D*	This study
GYQ179	*amyE::cat* P*xyl-sepF-G109K*	This study
GYQ180	*amyE::cat* P*xyl-sepF-F126S*	This study
3357	*ylmB-H::kan*	[9]
GYQ205	*amyE::cat* P*xyl-sepF-L7D sepF::erm*	GYQ178 transformed with GYQ134
GYQ206	*amyE::cat* P*xyl-sepF-G109K sepF::erm*	GYQ179 transformed with GYQ134
GYQ207	*amyE::cat* P*xyl-sepF-F126S sepF::erm*	GYQ180 transformed with GYQ134
GYQ185	*amyE::cat* P*xyl-sepF-F126S ylmB-H::kan*	GYQ215 transformed with 3357
GYQ223	*amyE::cat* P*xyl-sepF-F126S ylmB-H::kan yycH::erm*	GYQ185 transformed with GYQ201
GYQ224	*amyE::cat* P*xyl-sepF-F126S ylmB-H::kan yycI::erm*	GYQ185 transformed with GYQ202
GYQ210	*amyE::cat* P*xyl-sepF-F126S sepF::erm aprE*::(P*spac-ftsZ spec*)	GYQ207 transformed with TNVS101
PG62	*aprE::spec* P*spac-yfp-ftsA*	
GYQ33	*amyE::cat* P*xyl-sepF aprE::spec* P*spac-yfp-ftsA*	GYQ215 transformed with PG62
EKB36	*zapA:*(*Cm Pxyl-mcherry-zapA*)	Koning, unpub.
GYQ29	*zapA:*(*Kan Pxyl-mcherry-zapA*)	This study
GYQ212	*amyE::cat* P*xyl-sepF aprE::spec* P*spac-yfp-ftsA zapA:*(*Kan* P*xyl-mcherry-zapA*)	GYQ33 transformed with GYQ29
GYQ211	*amyE::cat* P*xyl-sepF ftsZ::spec ftsZ-gfp zapA:*(*Kan* P*xyl-mcherry-zapA*)	GYQ298 transformed with GYQ29
4057	*ezrA::Cm ezrA-gfp*	Laboratory stock
GYQ28	*ezrA::Kan ezrA-gfp*	This study
GYQ30	*amyE::cat* P*xyl-sepF ezrA::Kan ezrA-gfp*	GYQ215 transformed with GYQ28

E.coli

Top10		Laboratory stock

**Plasmid**	**Relevant features or genotype**	**Construction or reference**
pAPNC213 *Cm*	*bla, aprE3', Cm, lacI,* P*spac, aprE5'*	[10]
pAPNC213 *Erm*	*bla, aprE3', Erm, lacI,* P*spac, aprE5'*	[10]
pAPNC213 *Kan*	*bla, aprE3', Kan, lacI,* P*spac, aprE5'*	[10]
pMarB	*bla, erm* P*ctc Himar1 Kan* (TnYLB-1)	[11]
pMutin4	*bla, erm, lacI,* P*spac-lacZ*	[5]
pUC19	*bla,* P*lac*	[12]
pHJS105	*bla, amyE3', spec,* P*xyl-gfp-MCS, amyE5'*	[13]and H. Strahl
pEKC12	*bla, amyE3', spec,* P*xyl-gfp-pbpB, amyE5'*	This study
pEKC13	*bla, amyE3', spec,* P*xyl-gfp-ftsL, amyE5'*	This study
pEKC14	*bla, amyE3', spec,* P*xyl-gfp-ftsW, amyE5'*	This Study
pTNV9	*bla, aprE3', erm, lacI,* P*spac-gfp, aprE5'*	This study
pTNV42	*bla, Cm*	This study
pTNV60	*bla, Cm 3', Kan, Cm 5'*	This study
pYQ01	*bla, aprE3', erm, lacI,* P*spac-gfp-ftsW, aprE5'*	This study
pYQ02	*bla, aprE3', erm, lacI,* P*spac-gfp-ftsL, aprE5'*	This study
pYQ03	*bla, aprE3', erm, lacI,* P*spac-gfp-pbpB, aprE5'*	This study
pYQ05	*bla, erm, lacI,* P*ydcF-lacZ*	This study
pYQ10	*bla, amyE3', spec,* P*xyl-gfp-walK, amyE5'*	This study
pYQ11	*bla, aprE3', erm, lacI,* P*spac-gfp-walK, aprE5'*	This study
pYQ13	*bla, aprE3', Kan, lacI,* P*spac-walR, aprE5'*	This study
pYQ14	*bla, aprE3', Kan, lacI,* P*spac-walR R204C, aprE5'*	This study
pYQ40	*bla, erm, lacI,* P*accDA-lacZ*	This study
pYQ47	*bla, aprE3', spec,* P*srfAA-lacZ, aprE5'*	This study
pYQ56	*bla, aprE3', spec,* P*mtnK-lacZ, aprE5'*	This study
pYQ73	*bla, aprE3', spec,* P*rpsD-gfp, aprE5'*	This study
pYQ87	*bla, aprE3', spec, lacZ, aprE5'*	This study

pYQ141	*bla, aprE3', erm, lacI,* P*spac-mcherry-walK, aprE5'*	This study

Table S4: Primer sequences used in this study

Name	Sequence (5'-3')
YQ41	GCGCTCACAATTAGAAAGGAGATTCCTAGGATGG
YQ42	GGGCTAACGCCTAAATAGTACATAATGGATTTCCTT
YQ43	GTACTATTTAGGCGTTAGCCCAAGCGCATC
YQ44	TCCTTTCTAATTGTGAGCGCTCACAATTCCACA
YQ52	ATGGGGAAGAGAACCGCTTAAG
YQ53	ATGTTTGCAAAACGATTCAAAACC
YQ72	ACAGCGGAATTGACTCCACATTGTGAAATCTATTGAC
YQ73	CACAATGTGGAGTCAATTCCGCTGTCGATAACA
YQ75	GGCTAACGCCCGAATTCGAGCTCttaGTCCTGTTCTGGGTTTCTCA
YQ76	GAGCTCGAATTCGGGCGTTAG
YQ78	ACGTAACAGTCTGCCGGCTTCGTG
YQ79	CACGAAGCCGGCAGACTGTTACGT
YQ94	acgtaagcttACTCAACGTACCTGATATCCGCT
YQ95	TCATggatccTTTGAAATTGTCCTTTCAAAGAATC
YQ98	CTTCCAGATAACTGCCGTCACT
YQ99	AAGGCTCAGGAAGCGGCTCAATGAATAAGGTTGGTTTTTTTCGGT
YQ100	TGAGCCGCTTCCTGAGCCTTTGTAGAGCTCATCCATGCCAT
YQ101	AGCTTATCGATACCGTCGACTCACGCTTCATCCCAATCATC
YQ102	acgtAAGCTTCGATGGAAACAGGTGTTGCA
YQ103	tcatggatccATGATTACCTCCCTTTTGTGAA
YQ104	ATGAAGTGGCGAAGTTCACGA
YQ105	AAACTATGCGAGTGAAGACGTAGA
YQ142	tagGCAGGAGGAAAATCAAAATGatgGATAAAAAGATCCTTGTAGTAG
YQ143	CATTTTGATTTTCCTCCTGCctaGCAGGTCAATTGTGAGCGCTCACAATTCCAC
YQ149	GAGTATGAAAAATAAAGACAAAAACTTTTTCTCAATG
YQ150	CATTGAGAAAAAGTTTTTGTCTTTATTTTTCATACTC
YQ151	GACTTTTTAAGCAAGACCGTTTATGCCA
YQ152	TGGCATAAACGGTCTTGCTTAAAAAGTC
YQ153	TCGGCTCAGATATTTCCCTCTGCACGCCTGAC
YQ154	GTCAGGCGTGCAGAGGGAAATATCTGAGCCGA
YQ214	ATGGAAGTTACTGACGTAAGATTAC
YQ215	ATTTCAGTGCCGAATAGTCTGGA
YQ216	CTTACGTCAGTAACTTCCATCTTGACCACTTCACCCATAATTTC
YQ217	AAGCTTGTAGTTAAAGCTTTTTAGACATCTAATGCCCGGTTATTATTATTTTTGAC
YQ218	AAAGCTTTAACTACAAGCTTTTTAGACATCTAATGATATCGAATTCTAGTTCTAGAGC
YQ219	CAAAAGCCTAATTGAGAGAAGTTTCTATAGA
YQ220	CTTCTCTCAATTAGGCTTTTGTAAATTTGGAAAGTTACAC
YQ236	CAGACTATTCGGCACTGAAATGAAAGCCTCATGCCTATTCTTG
YQ237	TCTTACGTCAGTAACTTCCATATTGTCATACCTCCCCTAATCT
YQ246	AGACTATTCGGCACTGAAATAGGAAGGGCAAATCATTAAAGAGT
YQ247	TCTTACGTCAGTAACTTCCATAACCTCCAATTATGTAATTAATTAATATG
YQ450	CTAGGATGGGTACCCTGCAGATGGTCAGCAAGGGAGAGGA
YQ451	CTGCAGGGTACCCATCCTAGGAATC
YQ452	CCGGCTCAGGAAGCGGCTCAATGAATAAGGTTGGTTTTTTTCGGT
YQ453	TGAGCCGCTTCCTGAGCCGGATCCTGAGCCGCTTCCTGA
TerS117	TCGACTCTAGAGGATCCCCGGGT

TerS118	CCTGCAGGCATGCAAGCTTGGCGT
TerS125	CAAGCTTGCATGCCTGCAGGATGAACTTTAATAAAATTGATTTAGACAATTGGA
TerS126	CGGGGATCCTCTAGAGTCGAATAAAAGCCAGTCATTAGGCCT
TerS135	GGGCGTTAGCCCAAGCGCATCA
TerS136	GGTCAATTGTGAGCGCTCACAATTCCACA
TerS139	GTGAGCGCTCACAATTGACCGGGTACCCTGCAGATGAGCAAAGGA
TerS140	ATGCGCTTGGGCTAACGCCCCGCGGCCGCTCTAGAACTAGA
TerS257	TagGACTTCAAAGAGTTTTATGATTTATACCT
TerS258	cTAGTCATCCTTTACAGGAGTCCAAATACCA
TerS259	CTCCTGTAAAGGATGACTAgTGGTTTCAAAATCGGCTCCGTCGA
TerS260	TAAAACTCTTTGAAGTCctAACATCAGAGTATGGACAGTTGCGGA
TerS350	CACCGCCGACATTCGCGTGGCTCCA
TerS351	GCATCAGGGCTGCGGCATCCGGA
TerS352	GGGGCCAATAAACGGATTGTATTGT
TerS353	GCCTCTGCCCCTTGCAAATCGGATGCCT
EKP22	GTCGACGGTATCGATAAGCTTGAT
EKP30	GTGGATCCGAAGTCTGGACATTTT
EKP31	TGTCCAGACTTCGGATCCACatgATTCAAATGCCAAAAAA
EKP32	ATCAAGCTTATCGATACCGTCGACttaATCAGGATTTTTA
EKP33	TGTCCAGACTTCGGATCCACatgAGCAATTTAGCTTACCA
EKP34	AGCTTATCGATACCGTCGACtcaTTCCTGTATGTTTTTCA
EKP38	TGTCCAGACTTCGGATCCACatgTTAAAAAAAATGCTAAA
EKP39	AGCTTATCGATACCGTCGACttaCAGATAAACAGTTTTTT

References

1 Salzberg, L. I. *et al.* The WalRK (YycFG) and σ[I] RsgI regulators cooperate to control CwlO and LytE expression in exponentially growing and stressed *Bacillus subtilis* cells. *Molecular microbiology* **87**, 180-195 (2013).

2 Bisicchia, P. *et al.* The essential YycFG two-component system controls cell wall metabolism in *Bacillus subtilis*. *Molecular microbiology* **65**, 180-200 (2007).

3 Howell, A. *et al.* Genes controlled by the essential YycG/YycF two-component system of *Bacillus subtilis* revealed through a novel hybrid regulator approach. *Molecular microbiology* **49**, 1639-1655 (2003).

4 Fukuchi, K. *et al.* The essential two-component regulatory system encoded by *yycF* and *yycG* modulates expression of the *ftsAZ* operon in *Bacillus subtilis*. *Microbiology* **146**, 1573-1583 (2000).

5 Vagner, V., Dervyn, E. & Ehrlich, S. D. A vector for systematic gene inactivation in *Bacillus subtilis*. *Microbiology* **144**, 3097-3104 (1998).

6 Mercier, R., Kawai, Y. & Errington, J. Excess membrane synthesis drives a primitive mode of cell proliferation. *Cell* **152**, 997-1007 (2013).

7 Hamoen, L. W. & Errington, J. Polar Targeting of DivIVA in *Bacillus subtilis* Is Not Directly Dependent on FtsZ or PBP2B. *Journal of bacteriology* **185**, 693-697 (2003).

8 Gamba, P., Rietkötter, E., Daniel, R. A. & Hamoen, L. W. Tetracycline hypersensitivity of an *ezrA* mutant links GalE and TseB (YpmB) to cell division. *Frontiers in microbiology* **6** (2015).

9 Hamoen, L. W., Meile, J. C., de Jong, W., Noirot, P. & Errington, J. SepF, a novel FtsZ-interacting protein required for a late step in cell division. *Molecular microbiology* **59**, 989-999 (2006).

10 Morimoto, T. *et al.* Six GTP-binding proteins of the Era/Obg family are essential for cell growth in *Bacillus subtilis*. *Microbiology* **148**, 3539-3552 (2002).

11 Le Breton, Y., Mohapatra, N. P. & Haldenwang, W. G. *In vivo* random mutagenesis of *Bacillus subtilis* by use of TnYLB-1, a mariner-based transposon. *Applied and environmental microbiology* **72**, 327-333 (2006).

12 Yanisch-Perron, C., Vieira, J. & Messing, J. Improved M13 phage cloning vectors and host strains: nucleotide sequences of the M13mpl8 and pUC19 vectors. *Gene* **33**, 103-119 (1985).

13 Lewis, P. J. & Marston, A. L. GFP vectors for controlled expression and dual labelling of protein fusions in *Bacillus subtilis*. *Gene* **227**, 101-109 (1999).

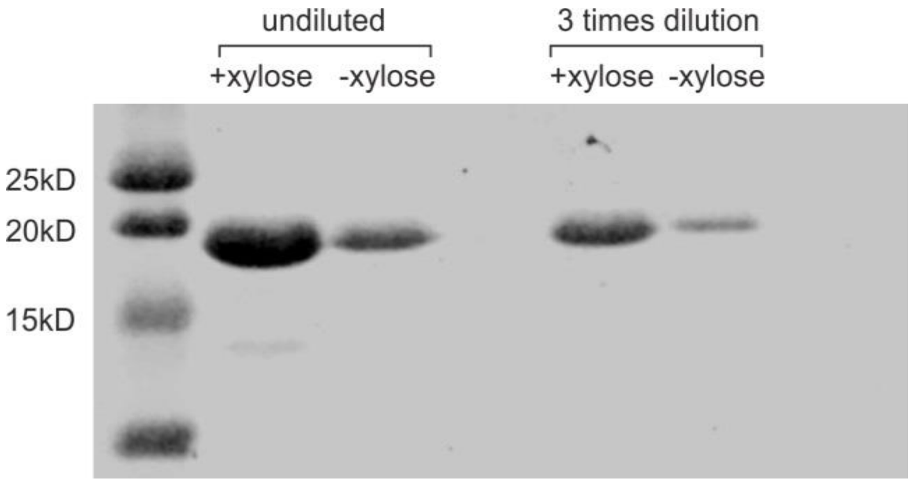

Fig. S1: SepF overexpression

Western blot analysis of SepF levels in SepF-overexpressing strain GYQ215 (*amyE::Pxyl-sepF*) grown in the presence (+) or absence (-) of 1% xylose for 3h. SepF primery antibody was used to detect the protein.

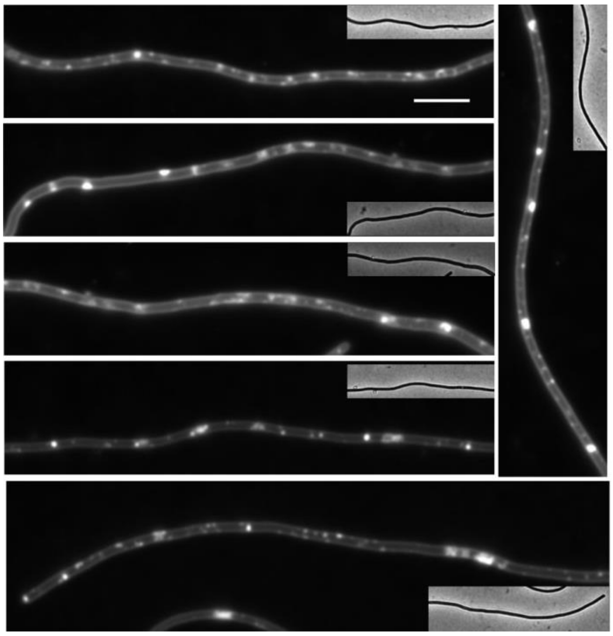

Fig. S2: Aberrant membrane invaginations

Microscopic images of strain GYQ215 (*amyE::Pxyl-sepF*) grown in presence of 1% xylose for 3h. Membranes were fluorescently stained with FM5-95.

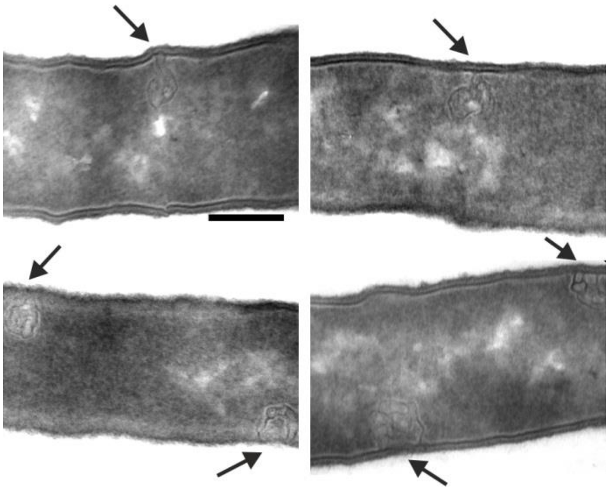

Fig. S3: Transmission electron microscopy

Transmission electron microscopy images of GYQ215 cells (*amyE::*P*xyl-sepF*) grown in LB with 1% xylose to overexpress SepF. Arrows indicate membrane invaginations. Scale bar is 200 nm.

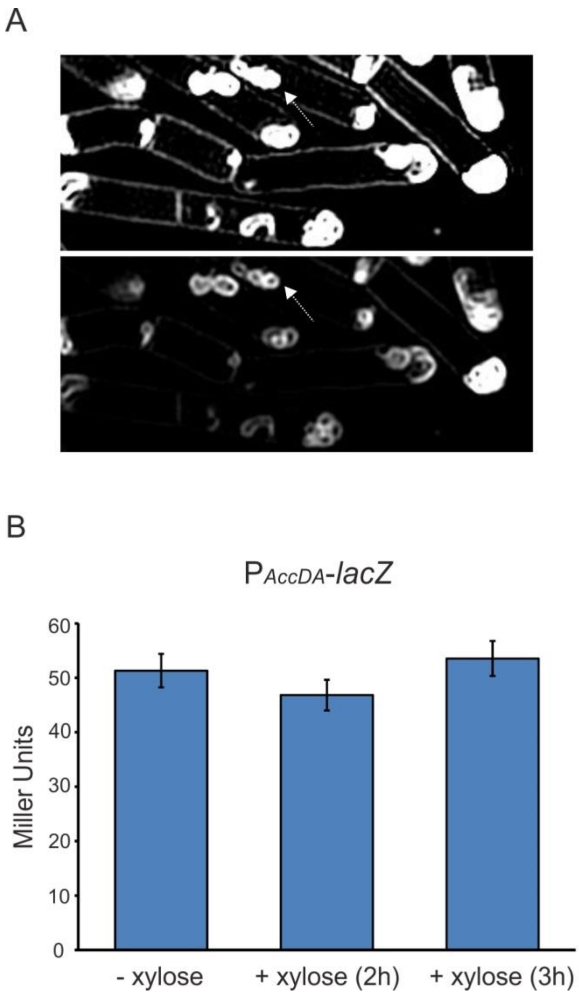

Fig. S4: High SepF levels do not induce AccDA expression

(A) N-SIM image of strain GYQ1738 (*amyE::*P*xyl-accDA*) grown with 1% xylose and stained with the membrane dye MitoTracker green showing the membrane invaginations. In the lower panel the brightness is reduced to better reveal membrane structures. (B) Effect of SepF overexpression on *accDA* promoter activity. β-galactosidase activities of P*accDA-lacZ* in the SepF overexpression strain (GYQ136, *amyE::*P*xyl-sepF* P*accDA-lacZ*) grown in either the absence or presence (SepF overexpression) of 1 % xylose for 2 and 3 h.

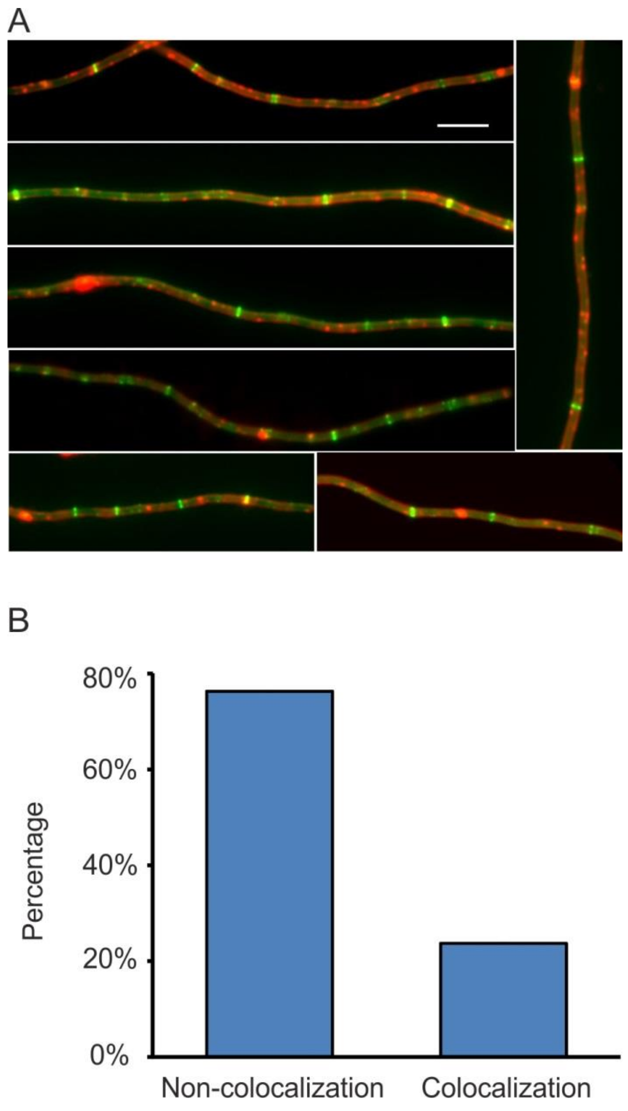

Fig. S5: Most Z-rings do not co-localize with membrane invaginations

(A) Microscopic images of strain GYQ298 (*amyE::*P*xyl-sepF ftsZ::ftsZ-gfp*) grown in the presence of 1% xylose for 3 h to overproduce SepF. Membranes were fluorescently stained with FM5-95. Scale bar is 5 µm. (B) Analysis of Z-rings that co-localized with membrane invaginations or not. In total 258 Z-rings were counted in 42 cells.

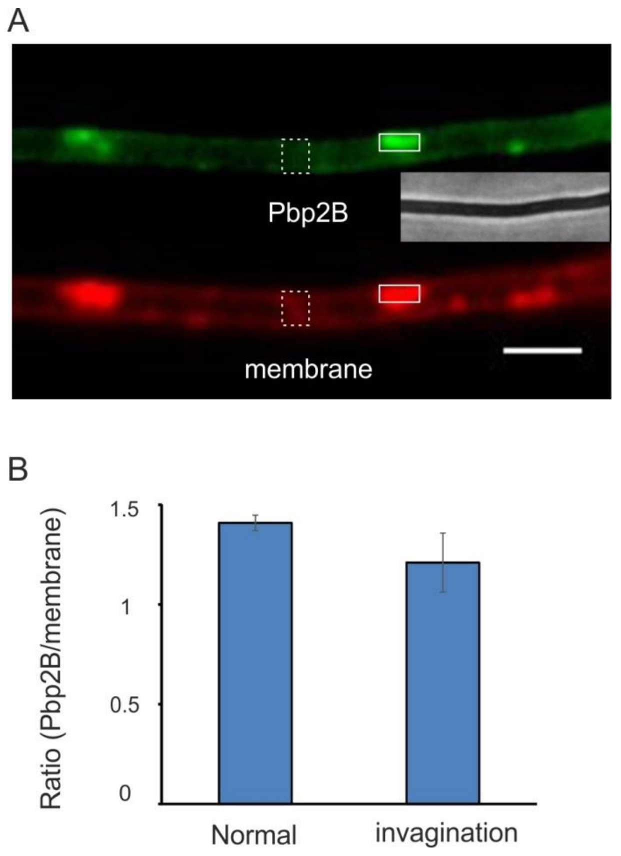

Fig. S6: Correlation of GFP-Pbp2B and FM5-95 fluorescence signals

Strain GYQ72 (*amyE::*P*xyl-sepF aprE::*P*spac-gfp-pbpB*) expressing GFP-Pbp2B) was grown with 1 % xylose for SepF overexpression and 5 mM IPTG for GFP-Pbp2B induction. Membranes were fluorescently stained with FM5-95. Scale bar is 2 µm. (A) White box with solid line indicates an invagination region selected for quantification of the GFP-Pbp2B and FM5-95 signals, and the box with dotted line shows a normal membrane region selected for quantification of the GFP-Pbp2B and FM5-95 signals. (B) Average ratio of GFP-Pbp2B and FM5-95 signals of 8 invaginations regions and 8 normal membrane regions.

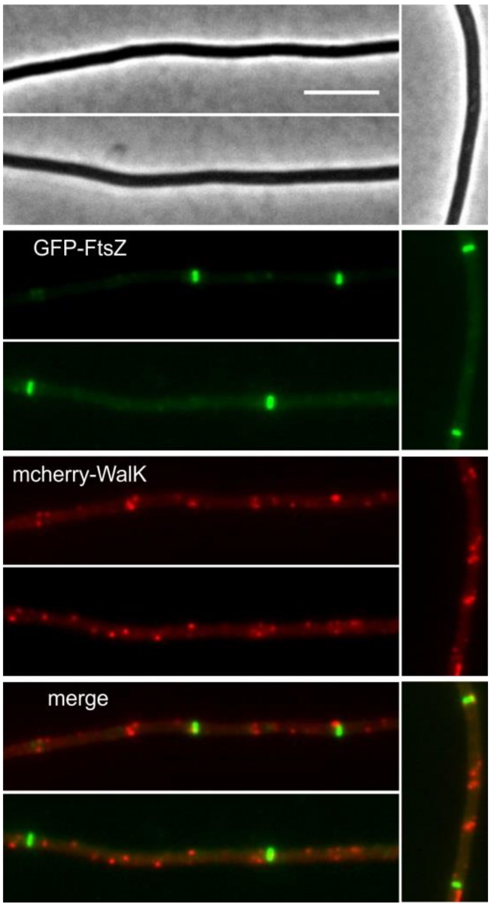

Fig. S7: WalK is delocalized from Z-rings

Microscopic images of strain GYQ571 (*amyE::*P*xyl-sepF ftsZ-gfp aprE::*P*spac-mCherry-walK*) expressing both FtsZ-GFP and mCherry-WalK in the presence of 1 % xylose to overexpress SepF. Scale bar is 5 µm.

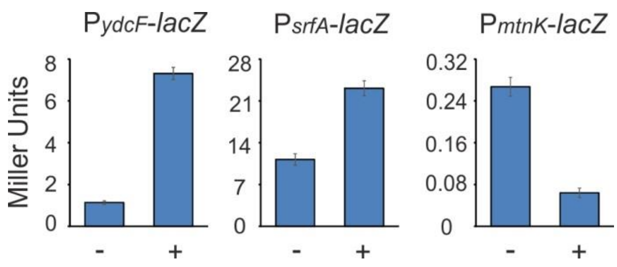

Fig. S8. Effect of SepF overexpression on gene expression

Transcriptional activity of promoters P*ydcF,* P*srfAA* and P*mtnK* in the absence (-) or presence (+) of extra SepF. SepF overexpression was achieved by growth in the presence of 1 % xylose. Transcription was measured using *lacZ* reporters (strain GYQ133, *amyE::*P*xyl-sepF* P*ydcF-lacZ,* strain GYQ199, *amyE::*P*xyl-sepF* P*sfrA-lacZ,* and strain GYQ218, *amyE::*P*xyl-sepF* P*mtnK-lacZ*, respectively).

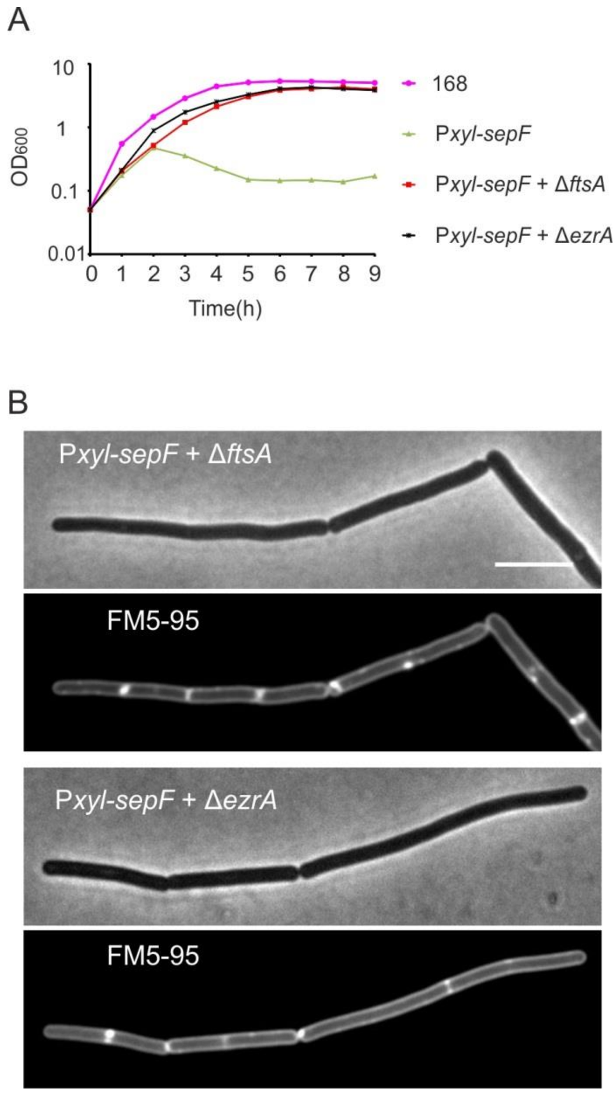

Fig. S9: Suppression of growth and cell division defect by deleting either *ezrA* or *ftsA*

(A) Growth of strains 168 (wild type), strain GYQ215 (*amyE::*P*xyl-sepF*), GYQ10 (*amyE::*P*xyl-sepF* Δ*ftsA*) and GYQ130 (*amyE::*P*xyl-sepF* Δ*ezrA*) in the presence of 1 % xylose (SepF overexpression). (B) Microscopic images of GYQ10 and GYQ130 after 3 h growth in medium with 1 % xylose. Membranes were stained with FM5-95. Scale bar is 5 µm.
